# In Vitro Faecal Fermentation of Monomeric and Oligomeric Flavan‐3‐ols: Catabolic Pathways and Stoichiometry

**DOI:** 10.1002/mnfr.202101090

**Published:** 2022-02-18

**Authors:** Giuseppe Di Pede, Letizia Bresciani, Furio Brighenti, Michael N. Clifford, Alan Crozier, Daniele Del Rio, Pedro Mena

**Affiliations:** ^1^ Human Nutrition Unit Department of Food and Drug University of Parma Via Volturno 39 Parma 43125 Italy; ^2^ School of Bioscience and Medicine Faculty of Health and Medical Sciences University of Surrey Guildford GU2 7XH UK; ^3^ Department of Nutrition Dietetics and Food School of Clinical Sciences at Monash Health Faculty of Medicine Nursing and Health Sciences Monash University BASE Facility, 264 Ferntree Gully Road Notting Hill Victoria 3168 Australia; ^4^ Department of Chemistry King Saud University Riyadh 11451 Saudi Arabia; ^5^ School of Medicine Dentistry and Nursing University of Glasgow Glasgow G12 8QQ UK; ^6^ Microbiome Research Hub University of Parma Parco Area delle Scienze 11/A Parma 43124 Italy

**Keywords:** human gut microbiota, phenolic catabolites, phenyl‐γ‐valerolactones, procyanidins, stoichiometry

## Abstract

**Scope:**

The study evaluates the influence of flavan‐3‐ol structure on the production of phenolic catabolites, principally phenyl‐γ‐valerolactones (PVLs), and phenylvaleric acids (PVAs).

**Methods and Results:**

A set of 12 monomeric flavan‐3‐ols and proanthocyanidins (degree of polymerization (DP) of 2–5), are fermented in vitro for 24 h using human faecal microbiota, and catabolism is analyzed by UHPLC‐ESI‐MS/MS. Up to 32 catabolites strictly related to microbial catabolism of parent compounds are detected. (+)‐Catechin and (−)‐epicatechin have the highest molar mass recoveries, expressed as a percentage with respect to the incubated concentration (75 µmol L^–1^) of the parent compound, for total PVLs and PVAs, both at 5 h (about 20%) and 24 h (about 40%) of faecal incubation. Only A‐type dimer and B‐type procyanidins underwent the ring fission step, and no differences are found in total PVL and PVA production (≃1.5% and 6.0% at 5 and 24 h faecal incubation, respectively) despite the different DPs.

**Conclusion:**

The flavan‐3‐ol structure strongly affects the colonic catabolism of the native compounds, influencing the profile of PVLs and PVAs produced in vitro. This study opens new perspectives to further elucidate the colonic fate of oligomeric flavan‐3‐ols and their availability in producing bioactive catabolites.

## Graphical abstract

Monomeric and oligomeric flavan‐3‐ols occur widely in our diet. This study involved a 24 h in vitro faecal fermentation of flavan‐3‐ols by human gut microbiota to assess the role of the degree of polymerization, A/B‐linkage type and galloyl moieties on microbiota‐derived catabolite production. Up to 32 colonic catabolites, directly related to the microbial catabolism of parent compounds, were identified. Catabolite pathways and stoichiometric balances in their production were highlighted.

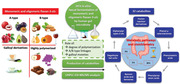

## 1 Introduction

Flavan‐3‐ols are the most consumed flavonoids in the western diet,^[^ ^1^, ^2^, ^3^, ^4^ ^]^ with green tea, red wine, dark chocolate, and some fruits and nuts as the main contributors to their daily intake. Flavan‐3‐ols range from simple monomers to polymeric proanthocyanidins (PACs), also known as condensed tannins. Procyanidins (PCs) are exclusively composed of (epi)catechin units. B‐type PCs have C_4_−C_8_ and/or C_4_−C_6_ linkages, while A‐type PCs contain an additional ether linkage between the C_2_ in the B‐ring of the adjacent unit and the oxygen‐bearing C_7_ in the A‐ring of the terminal (epi)catechin unit.^[^ ^5^ ^]^

After ingestion, monomeric flavan‐3‐ols are rapidly absorbed in the upper gastro‐intestinal tract, while up to the 90% of the ingested PACs reach the colon intact where they become substrates for microbial breakdown.^[^ ^6^ ^]^ The dietary intake of flavan‐3‐ols has been associated with several beneficial effects on human health,^[^ ^7^, ^8^ ^]^ and this is associated not with their native structure, but with the bioactivity of their circulating metabolites and catabolites,^[^ ^5^, ^9^, ^10^, ^11^, ^12^, ^13^, ^14^, ^15^ ^]^ among which phenyl‐γ‐valerolactones (PVLs) and phenylvaleric acids (PVAs) represent the most important C_6_–C_5_ catabolites.^[^ ^5^ ^]^ These catabolites are specific to flavan‐3‐ols^[^ ^5^ ^]^ and they exhibited various bioactive properties,^[^ ^16^, ^17^, ^18^ ^]^ becoming plausible candidates responsible for the recognized biological activity attributed to the intake of their parent compounds. There are reports that the A‐type dimer is more bioactive than its B‐type counterpart^[^ ^19^ ^]^ and that the bioactivity at a systemic level tends to be stronger with the increase in the degree of polymerization (DP).^[^ ^20^, ^21^ ^]^ However, these studies take into account neither the negligible absorption of the native oligomeric structures,^[^ ^6^ ^]^ nor their colonic catabolism.^[^ ^5^, ^22^, ^23^ ^]^ The structural properties of parent flavan‐3‐ol monomers and PACs may affect the native compound‐colonic microflora interaction and could represent a key aspect of the beneficial properties associated with the intake of these phytochemicals. The catabolism of PCs has been investigated with in vivo^[^ ^24^, ^25^, ^26^ ^]^ and in vitro studies,^[^ ^27^, ^28^, ^29^ ^]^ and the production of PVLs from B‐type dimers as well as from PCs contained in complex matrices such as nuts and fruits, has been well‐established.^[^ ^30^, ^31^, ^32^, ^33^, ^34^, ^35^, ^36^ ^]^ However, less is known about the interaction of the human gut microbiota with A‐type dimers,^[^ ^27^ ^]^ the ability of the microbiota to catabolize A‐ and/or B‐type oligomers, and the possible production of PVLs and PVAs from these flavan‐3‐ol structures.

The aim of this study was to elucidate the influence of flavan‐3‐ol structure, including DP, different subunit linkages (A‐/B‐type), and the presence of galloyl moieties, on the production of catabolites, in particular PVLs and PVAs. Molar mass recoveries of gut microbiota catabolites were calculated to estimate their contribution to the colonic degradation of parent flavan‐3‐ols. The study also aimed at defining stoichiometric balances in the production of PVLs and PVAs to estimate the dose of parent compounds to be ingested to achieve a known amount of circulating 5‐carbon side chain ring fission catabolites (5C‐RFC s). Twelve flavan‐3‐ols were incubated individually with human faecal microbiota for 5 and 24 h and the resultant catabolites were analyzed by UHPLC‐ESI‐MS/MS.

## 2 Experimental Section

### 2.1 Chemicals and Reagents

Formic acid, bile salts, soluble starch, (+)‐arabinogalactan, tryptone, yeast extract, xylan from birchwood, L‐cysteine hydrochloride monohydrate, guar gum, inulin, Tween 80, buffered peptone water, Dulbecco's phosphate buffer saline (PBS), casein sodium salt from bovine milk, pectin from citrus fruits, mucin from porcine stomach‐type III, CaCl_2_, KCl, NaCl, NaHCO_3_, anhydrous K_2_HPO_4_, KH_2_PO_4_, MgSO_4_ monohydrate, FeSO_4_ heptahydrate, resazurin redox indicator, (+)‐catechin, (−)‐epicatechin, phenylacetic acid, 4′‐hydroxyphenylacetic acid, 3′‐hydroxyphenylacetic acid, 3′,4′‐dihydroxyphenylacetic acid, 3‐phenylpropanoic acid, 3‐(4′‐hydroxyphenyl)propanoic acid, 3‐(3′‐hydroxyphenyl)propanoic acid, 3‐(3′,4′‐dihydroxyphenyl)propanoic acid, benzoic acid, 4‐hydroxybenzoic acid, 3‐hydroxybenzoic acid, 3,4‐dihydroxybenzoic acid, 3,4,5‐trihydroxybenzoic acid, benzene‐1,2,3‐triol, benzene‐1,3,5‐triol, 3,4‐dihydroxybenzaldehyde, and 4‐hydroxybenzaldehyde were purchased from Sigma‐Aldrich (St Louis, MO, USA). 5‐(4′‐Hydroxyphenyl)‐valerolactone, 5‐(3′‐hydroxyphenyl)‐valerolactone, 5‐(3′,4′‐dihydroxyphenyl)‐γ‐valerolactone, 5‐(3′,5′‐dihydroxyphenyl)‐γ‐valerolactone and 5‐(3′,4′,5′‐trihydroxyphenyl)‐γ‐valerolactone were synthesized in house.^[^ ^37^ ^]^ 5‐(4′‐Hydroxyphenyl)valeric acid was purchased from Toronto Research Chemicals (Toronto, ON, Canada). (−)‐Epigallocatechin‐3‐*O*‐gallate (EGCG), dimer A2, dimer B2 and trimer BB were purchased from Extrasynthese (Genay Cedex, France). Trimer AA, trimer AB, tetramer ABA, tetramer BBB and pentamer BBBB were purchased from PlantaAnalytica (New Milford, CT, USA). Theaflavin‐3′‐*O*‐gallate was purchased from LGC STANDARD (Milan, Italy). Oligomers were checked for possible monomeric flavan‐3‐ol content by UHPLC‐ESI‐MS/MS analysis, resulting below 1%, w/w. All solvents and reagents were UHPLC‐grade and were purchased from VWR International (Milan, Italy), unless otherwise indicated. Ultrapure water from MilliQ system (Millipore, Bedfort, MA, USA) was used throughout the experiment.

### 2.2 In Vitro Faecal Fermentation Substrates

Twelve flavan‐3‐ols, including the monomers (+)‐catechin and (−)‐epicatechin, oligomeric PACs with a different DP, including two dimers [dimer A2, and dimer B2], three trimers [trimer AA, trimer AB, and trimer BB], two tetramers [tetramer ABA, and tetramer BBB], and one pentamer, and two galloyl derivatives, namely EGCG and theaflavin‐3′‐*O*‐gallate, were employed individually as substrates for microbial catabolism through in vitro faecal fermentations. Structural properties of these compounds are detailed in **Table** **1**.

**Table 1:** Structural properties of monomeric and oligomeric flavan‐3‐ols used as substrates for microbial breakdown

Compound	DP	Monomeric units	Linkage type	Galloyl units
(+)‐catechin	1	–	–	–
(−)‐epicatechin	1	–	–	–
Dimer A2	2	(−)‐epicatechin, (+)‐epicatechin	A type	–
Dimer B2	2	(−)‐epicatechin, (−)‐epicatechin	B type	–
Trimer AA	3	(−)‐epicatechin, (−)‐epicatechin, (−)‐catechin	A type, A type	–
Trimer AB	3	(−)‐epicatechin, (−)‐epicatechin, (−)‐catechin	A type, B type	–
Trimer BB	3	(−)‐epicatechin, (−)‐epicatechin, (−)‐epicatechin	B type, B type	–
Tetramer ABA	4	(−)‐epicatechin, (+)‐epicatechin, (−)‐epicatechin, (−)‐catechin	A type, B type, A type	–
Tetramer BBB	4	(−)‐epicatechin, (−)‐epicatechin, (−)‐epicatechin, (−)‐epicatechin	B type, B type, B type	–
Pentamer BBBB	5	(−)‐epicatechin, (−)‐epicatechin, (−)‐epicatechin, (−)‐epicatechin, (−)‐epicatechin	B type, B type, B type, B type	–
(−)‐Epigallocatechin‐3‐*O*‐gallate	1	–	‐	1
Theaflavin‐3′‐*O*‐gallate	2	–	‐	1

DP = degree of polymerization.

### 2.3 In Vitro Colonic Biotransformation Procedure

Growth medium and faecal slurries were prepared, and the in vitro incubations performed as described previously.^[^ ^38^, ^39^, ^40^, ^41^ ^]^ Briefly, 1 L of growth medium was prepared, aliquoted, and sterilized at 121 °C for 15 min in glass vessels (12 mL) before sample preparation. Fresh faeces were collected from three healthy volunteers (2 women and 1 man, aged 24.0 ± 5.6, height 1.7 ± 0.1 m, weigh 66.3 ± 15.5 kg, and BMI 21.8 ± 2.9 kg m^–2^ (mean ± SD)) who did not have any intestinal disease and were not treated with antibiotics for the previous 3 months.^[^ ^38^ ^]^ Donors followed a controlled diet lacking (poly)phenol‐containing foods for 2 days prior to faecal collection. After collection, faeces were immediately stored in an anaerobic jar and processed within 2 h. Faeces from donors were pooled in equal amount and homogenized with 1% w/v sterilized PBS to obtain a 10% w/v faecal slurry used as fermentation starter.^[^ ^38^ ^]^ The Ethics Committee of Area Vasta Emilia Nord (AVEN) approved the collection and use of the faecal slurries (protocol no. 796/2018/sper/unipr) and all the donors provided informed consent for the collection of faecal slurries.

Parent compounds were dissolved in an aqueous bile salt solution^[^ ^42^ ^]^ and suspensions were left for 2 h at room temperature under constant magnetic stirring.^[^ ^40^ ^]^ In each fermentation batch, 45% of the growth medium, 45% of faecal slurry, and 10% of substrate suspension were added to reach a total fermentation volume of 4 mL.^[^ ^43^ ^]^ The faecal slurry and the aqueous product suspension were put into the vessel containing growth medium, were sealed, and flushed with N_2_ to create anaerobiosis. Parent compounds were fermented at a final concentration of 75 µmol L^–1^. This concentration is in line with the concentration of flavan‐3‐ols found in the ileal fluid of ileostomy patients consuming dietary amounts of flavan‐3‐ols.^[^ ^6^, ^44^, ^45^ ^]^ Blank samples containing the growth medium and the faecal slurry, without substrate aqueous suspension, as well as abiotic control samples, containing the growth medium and the substrate suspensions without faecal starter, were also prepared.^[^ ^43^ ^]^ Vessels were incubated for 24 h at 37 °C at 200 strokes min^–1^ in a Dubnoff bath (JULABO, Seelbach Germany). Samples were collected prior starting the fecal fermentation (0 h) and after 5 h and 24 h incubation. Microbial catabolism was stopped adding 10% v/v of acetonitrile,^[^ ^40^, ^41^ ^]^ and samples were frozen (−20 °C) until extraction and analysis. All experiments were carried out in triplicate.

### 2.4 Fermented Sample Preparation

Different solvents, including I) acidified methanol (0.1% v/v formic acid),^[^ ^46^ ^]^ II) acidified acetonitrile (0.1% v/v formic acid),^[^ ^47^ ^]^ III) acidified ethyl acetate (0.1% v/v formic acid)^[^ ^30^, ^40^ ^]^) and a Solid Phase Extraction (SPE) method^[^ ^48^ ^]^ were employed to determine the extraction efficiency from fermented samples for both parent compounds and their related gut microbiota catabolites, adopting the method of Di Pede et al.^[^ ^40^ ^]^ with minor modifications. Based on these preliminary analyses, acidified methanol (0.1% v/v formic acid) displayed the best extraction recovery for the analytes. Briefly, an aliquot of 300 µL of each fermented sample was finally extracted with acidified methanol (0.1% v/v formic acid) (1.2 mL), vortexed for 30 s, sonicated for 10 min in an ultrasonic bath, vortexed for 30 s, and re‐sonicated for 5 min. Samples were centrifuged (Centrisart A‐14C Refrigerated Micro‐Centrifuge and Rotor YCSR‐A1C, Sartorius Lab Instruments GmbH and Co. KG, Goettingen, Germany) at 14 460 × *g* for 10 min and the upper organic layer was transferred into a clean microfuge tube. After the first extraction, the residual pellet of the fermented samples was re‐extracted following the same procedure, using 500 µL of the same solvent. Finally, supernatants were pooled, vortexed for 30 s and centrifuged at 14 460 × *g* for 10 min before UHPLC‐ESI‐MS/MS analysis.

### 2.5 UHPLC‐ESI‐MS/MS Analysis

Samples were analyzed using a UHPLC DIONEX Ultimate 3000 fitted with a TSQ Vantage triple quadrupole mass spectrometer (MS) (Thermo Fisher Scientific Inc., San Jose, CA, USA) equipped with a heated‐electrospray ionization source (H‐ESI‐II; Thermo Fisher Scientific Inc.). Chromatographic and ionization parameters were based on the method of Brindani et al.^[^ ^37^ ^]^ with slight modifications for UHPLC conditions: mobile phase A was 0.01% v/v formic acid in water and mobile phase B was acetonitrile containing 0.01% v/v formic acid. Substrates and their catabolites were monitored in the SRM mode. The evaluation of the range of calibration curves was based on data fitting to linear regression, and acceptable fitting was estimated by using the coefficient of determination (R^2^). The limit of detection (LOD) and limit of quantification (LOQ) for reference standards were determined as the concentration in which the quantifier transition showed a signal‐to‐noise (S/N) ratio ≥ 3 and ≥ 10, respectively (Table S1, Supporting Information). Chromatograms and mass spectral data were acquired using Xcalibur software 2.1 (Thermo Fisher Scientific Inc.). Parent compounds were quantified by using calibration curves of respective standards in a concentration range of 0.05–10.0 µmol L^–1^. When standards were not available catabolites were quantified by using calibration curves of structurally similar compounds, in a concentration range of 0.02–75.0 µmol L^–1^.

### 2.6 Data and Statistical Analysis

Results were presented as mean values ± SD. In accordance with previous studies,^[^ ^31^, ^34^ ^]^ molar mass recoveries for catabolites were expressed as percentage (%) with respect to the incubated concentration of parent compound (75 µmol L^–1^) to facilitate comparisons among the different fermented products. Molar mass recoveries in the production of total 5C‐RFCs (PVLs and PVAs) and total catabolites were calculated as the sum of single molar mass recovery of 5C‐RFCs and total catabolites, respectively. Stoichiometric balances in the production of 5C‐RFCs (PVLs and PVAs) were estimated through molar mass recoveries assuming the production of PVLs and PVAs i) from 1 µmol of incubated parent compound, or ii) from 1 µmol of all the possible monomeric unit released from the oligomeric structure in accordance with the DP (2‐5) of parent compound. General Linear Models (GLM) were used to evaluate i) the effect of time, treatment, and treatment of the incubation process (treatment × time) on molar mass recovery of monomeric and dimeric units, diphenylpropan‐2‐ols, PVLs, PVAs, 3‐(phenyl)propanoic acid, benzoic acid, and benzene derivatives produced from parent compounds during the faecal incubations, ii) the effect of time on molar mass recovery of catabolites produced from fission of parent compounds with a DP >1, iii) the effect of time, treatment, and treatment × time on molar mass recoveries of total 5C‐RFCs (PVLs and PVAs) and total catabolites.

ANOVA with Tukey's post hoc test was applied to detect differences in molar mass recoveries of the catabolites produced from faecal incubations with different substrates over the same incubation period (T5 and T24). *t*‐Test was applied to compare data on molar mass recoveries of catabolites considering the same fermented substrate for different incubation periods (T5 and T24), and for benzene derivatives produced from EGCG incubation (T5 vs T24) and from fermentation of EGCG versus theaflavin‐3′‐*O*‐gallate at 24 h. A difference was considered significant at *p* < 0.05.

PCA with varimax rotation was applied to explore differences in the behavior of parent compounds in the in vitro colonic environment and in the appearance of catabolites over the fecal fermentation. PCA was carried out taking into account the single concentrations of all the catabolites produced over time, with the exception of fission catabolites and theaflavin derived from the degradation of parent compounds having a DP >1 and from catabolism of theaflavin‐3′‐*O*‐gallate, respectively. All statistical analyses were performed with the SPSS statistical software (version 26, SPSS, Inc., Chicago, IL, USA).

## 3 Results

### 3.1 Degradation of Substrates and Identification of Microbial Catabolites

Twelve substrates comprising monomeric flavan‐3‐ols and PACs (DP 2–5) were incubated with faecal material for 5 and 24 h at a concentration of 75 µmol L^–^.^1^ After 5 h faecal fermentation, the decrease of parent compounds ranged from 17% to 100% for trimer AA and (+)‐catechin, respectively, and from 33% to >95% for theaflavin‐3′‐*O*‐gallate and for the remaining substrates, respectively, after 24 h. Tetramer ABA was not catabolized by colonic microbiota over the 24 h‐in vitro incubation.

**Figure 1:**
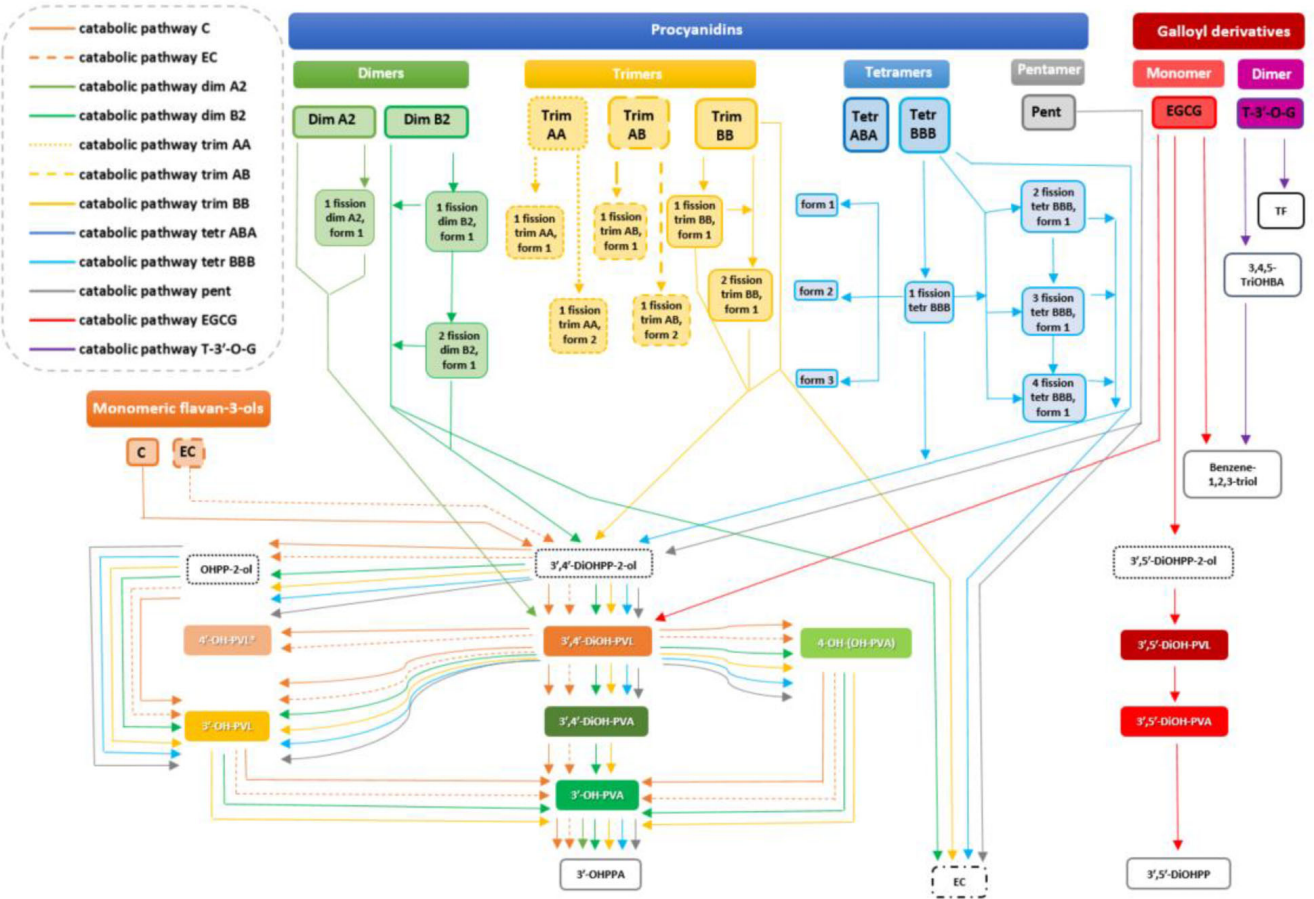
Proposed catabolic pathways and catabolites produced after in vitro faecal fermentation of monomeric and oligomeric flavan‐3‐ols. C: (+)‐catechin; EC: (−)‐epicatechin; Dim: dimer; Trim: trimer; Tetr: tetramer; Pent: pentamer; EGCG: (−)‐epigallocatechin‐3‐*O*‐gallate; T‐3′‐*O*‐G: theaflavin‐3′‐*O*‐gallate; OHPP‐2‐ol: 1‐(hydroxyphenyl)‐3‐(2″,4″,6″‐trihydroxyphenyl)‐propan‐2‐ol; 3′,5′‐DiOHPP‐2‐ol: 1‐(3′,5′‐dihydroxyphenyl)‐3‐(2″,4″,6″‐trihydroxyphenyl)‐propan‐2‐ol; 3′,4′‐DiOHPP‐2‐ol: 1‐(3′,4′‐dihydroxyphenyl)‐3‐(2″,4″,6″‐trihydroxyphenyl)‐propan‐2‐ol; 3′‐OH‐PVL: 5‐(3′‐hydroxyphenyl)‐γ‐valerolactone; 4′‐OH‐PVL: 5‐(4′‐hydroxyphenyl)‐γ‐valerolactone; 3′,5′‐DiOH‐PVL: 5‐(3′,5′‐dihydroxyphenyl)‐γ‐valerolactone; 3′,4′‐DiOH‐PVL: 5‐(3′,4′‐dihydroxyphenyl)‐γ‐valerolactone; 3′‐OH‐PVA: 5‐(3′‐hydroxyphenyl)valeric acid; 4‐OH‐(OH‐PVA): 4‐hydroxy‐5‐(hydroxyphenyl)valeric acid; 3′,5′‐DiOH‐PVA: 5‐(3′,5′‐dihydroxyphenyl)valeric acid; 3′,4′‐DiOH‐PVA: 5‐(3′‐4′‐dihydroxyphenyl)valeric acid; 3′‐OHPPA: 3‐(3′‐hydroxyphenyl)propanoic acid; 3′,5′‐DiOHPPA: 3‐(3′,5′‐dihydroxyphenyl)propanoic acid; 3,4,5‐TriOHBA: 3,4,5‐trihydroxybenzoic acid; TF: theaflavin. * means not quantified in fermented samples.

A total of 105 compounds were monitored by UHPLC‐ESI‐MS/MS (Table S2, Supporting Information) and 32 catabolites were identified and quantified in the various samples following faecal fermentation. These comprised mainly diphenylpropan‐2‐ols, PVLs and their related PVAs, and 3‐(phenyl)propanoic acids (**Table** **2**). Retention time and selective reaction monitoring (SRM) conditions employed for identification and quantification of compounds are reported in Table S2, Supporting Information. In absence of available standards for some catabolites, the criteria for identification were based on previously reported LC‐MS analyses.^[^ ^5^, ^30^, ^34^, ^37^, ^41^, ^49^, ^50^ ^]^ In the control samples, consisting of growth medium and faecal material without flavan‐3‐ols substrates, or substrates and growth medium without the faecal starter, no more than negligible amounts of catabolites were detected, indicating that the (poly)phenol‐free diet produced almost blank faeces, and that the incubation process did not influence the production of the quantified catabolites. A potential pathway of each in vitro fermented parent compounds is presented in **Figure** **1**, indicating possible routes involved in the gut microbiota‐mediated catabolism.

**Table 2:** Gut microbiota catabolites identified after 5 and 24 h faecal fermentation of monomeric and oligomeric flavan‐3‐ols

Molecular weight (Da)	Gut microbiota catabolites
	Fission catabolites of oligomers
	Dimers
	*Derived from catabolic pathway of dimer A2*
578	1 fission dimer A2, form 1
	*Derived from catabolic pathway of dimer B2*
580	1 fission dimer B2, form 1
582	2 fission dimer B2, form 1
	Trimers
	*Derived from catabolic pathway of trimer AA*
864	1 fission trimer AA, form 1
864	1 fission trimer AA, form 2
	*Derived from catabolic pathway of trimer AB*
866	1 fission trimer AB, form 1
866	1 fission trimer AB, form 2
	*Derived from catabolic pathway of trimer BB*
868	1 fission trimer BB, form 1
870	2 fission trimer BB, form 1
	Tetramers
	*Derived from catabolic pathway of tetramer BBB*
1156	1 fission tetramer BBB, form 1
1156	1 fission tetramer BBB, form 2
1156	1 fission tetramer BBB, form 3
1158	2 fission tetramer BBB, form 1
1160	3 fission tetramer BBB, form 1
1162	4 fission tetramer BBB, form 1
	Diphenylpropan‐2‐ols
276	1‐(Hydroxyphenyl)‐3‐(2″,4″,6″‐trihydroxyphenyl)‐propan‐2‐ol
292	1‐(3′,5′‐Dihydroxyphenyl)‐3‐(2″,4″,6″‐trihydroxyphenyl)‐propan‐2‐ol
292	1‐(3′,4′‐Dihydroxyphenyl)‐3‐(2″,4″,6″‐trihydroxyphenyl)‐propan‐2‐ol
	Phenyl‐γ‐valerolactones
192	5‐(4′‐Hydroxyphenyl)‐γ‐valerolactone
192	5‐(3′‐Hydroxyphenyl)‐γ‐valerolactone
208	5‐(3′,5′‐Dihydroxyphenyl)‐γ‐valerolactone
208	5‐(3′,4′‐Dihydroxyphenyl)‐γ‐valerolactone
	Phenylvaleric acids
194	5‐(3′‐Hydroxyphenyl)valeric acid
210	4‐Hydroxy‐5‐(hydroxyphenyl)valeric acid
210	5‐(3′,5′‐Dihydroxyphenyl)valeric acid
210	5‐(3′,4′‐Dihydroxyphenyl)valeric acid
	Phenylpropanoic acids
166	3‐(3′‐Hydroxyphenyl)propanoic acid
182	3‐(3′,5′‐Dihydroxyphenyl)propanoic acid
	Benzoic acid
170	3,4,5‐Trihydroxybenzoic acid
	Benzene derivative
126	Benzene‐1,2,3‐triol
	Monomer unit
290	(−)‐Epicatechin
	Dimer unit
564	Theaflavin

The nomenclature of catabolites was standardized according to Kay et al.[60] Chromatographic and spectrometric information is provided in Table S2, Supporting Information. Structures of fission derivatives of dimer A2 and dimer B2 are reported in Figures S2 and S3, Supporting Information.

### 3.2 Molar Mass Recoveries for Catabolites

The main effects of treatment, time and treatment × time (*p* < 0.001) were observed for the production of diphenylpropan‐2‐ols [1‐(hydroxyphenyl)‐3‐(2″,4″,6″‐trihydroxyphenyl)‐propan‐2‐ol, 1‐(3′,5′‐dihydroxyphenyl)‐3‐(2″,4″,6″‐trihydroxyphenyl)‐propan‐2‐ol, 1‐(3′,4′‐dihydroxyphenyl)‐3‐(2″,4″,6″‐trihydroxyphenyl)‐propan‐2‐ol], PVLs [5‐(3′‐hydroxyphenyl)‐γ‐valerolactone, 5‐(3′,5′‐dihydroxyphenyl)‐γ‐valerolactone, 5‐(3′,4′‐dihydroxyphenyl)‐γ‐valerolactone], PVAs [5‐(3′‐hydroxyphenyl)valeric acid, 4‐hydroxy‐5‐(hydroxyphenyl)valeric acid), 5‐(3′,5′‐dihydroxyphenyl)valeric acid, 5‐(3′,4′‐dihydroxyphenyl)valeric acid], 3‐(3′‐hydroxyphenyl)propanoic acid and 3‐(3′,5′‐dihydroxyphenyl)propanoic acid, benzoic acid and benzene derivatives [3,4,5‐trihydroxybenzoic acid and benzene‐1,2,3‐triol], (−)‐epicatechin and theaflavin (**Table** **3**). A substantial significant effect of time (*p* < 0.001) was also observed for the production of oligomers with interflavanic linkage fission and/or C‐ring opening (**Table** **4**).

**Table 3:** Molar mass recoveries (%) for diphenylpropan‐2‐ols, phenyl‐γ‐valerolactones (PVLs), phenylvaleric acids (PVAs), phenylpropanoic acids, benzoic acid and benzene derivatives, (−)‐epicatechin (EC) and theaflavin (TF) produced after 5 and 24 h faecal fermentation of monomeric and oligomeric flavan‐3‐ols

Substrates	Time [h]	EC	OHPP‐2‐ol	3′,5′‐DiOHPP‐2‐ol	3′,4′‐DiOHPP‐2‐ol	3′‐OH‐PVL	3′,5′‐DiOH‐PVL	3′,4′‐DiOH‐PVL	3′‐OH‐PVA	4‐OH‐(OH‐PVA)	3′,5′‐DiOH‐PVA	3′,4′‐DiOH‐PVA	3′‐OHPPA	3′,5′‐DiOHPPA	3,4,5‐TriOHBA	Benzene‐1,2,3‐triol	TF
Flavan‐3‐ol monomers																	
(+)‐Catechin	5	n.d.	44.0 ± 2.0^a^	‐	0.2 ± 0.1^d^	1.5 ± 0.3^b^	‐	6.1 ± 0.6^b;***^	n.d.	0.2 ± 0.1	‐	n.d.	4.5 ± 0.2^a^	‐	‐	‐	‐
	24	n.d.	n.d.	‐	n.d.	17.5 ± 5.3^a;***^	‐	1.3 ± 0.5^b,c^	1.1 ± 0.1^b^	8.7 ± 0.7^b;**^	‐	0.3 ± 0.1^b,c^	15.5 ± 1.6^d,e;***^	‐	‐	‐	‐
(−)‐Epicatechin	5	n.d.	22.4 ± 2.2^b^	‐	12.6 ± 0.8^a^	4.7 ± 0.8^a^	‐	14.9 ± 1.4^a;**^	n.d.	0.7 ± 0.1	‐	n.d.	4.4 ± 0.3^a^	‐	‐	‐	‐
	24	n.d.	n.d.	‐	n.d.	17.9 ± 15.3^a^	‐	5.0 ± 2.9^a^	2.7 ± 1.0^a^	12.9 ± 2.1^a;**^	‐	1.7 ± 0.4^a^	12.0 ± 4.3^d,e;*^	‐	‐	‐	‐
Flavan‐3‐ol																	
dimers																	
Dimer A2	5	n.d.	n.d.	‐	n.d.	n.d.	‐	0.2 ± 0.0^d,e^	n.d.	n.d.	‐	n.d.	0.9 ± 0.3^c^	‐	‐	‐	‐
	24	n.d.	n.d.	‐	n.d.	n.d.	‐	0.3 ± 0.1^c;*^	n.d.	n.d.	‐	n.d.	10.5 ± 3.6^e;**^	‐	‐	‐	‐
Dimer B2	5	2.5 ± 1.4^a^	1.5 ± 0.0^c;***^	‐	4.4 ± 2.6^b^	n.d.	‐	1.3 ± 1.3^c,d,e^	n.d.	n.d.	‐	n.d.	1.8 ± 0.5^b^	‐	‐	‐	‐
	24	n.d.	0.1 ± 0.0^c^	‐	0.1 ± 0.0^b^	5.4 ± 0.4^b^	‐	6.3 ± 0.5^a;**^	0.1 ± 0.0^c^	1.7 ± 0.2^c,d^	‐	0.2 ± 0.0^c^	43.5 ± 1.1^b;***^	‐	‐	‐	‐
Trimers																	
Trimer AA	5	n.d.	n.d.	‐	n.d.	n.d.	‐	n.d.	n.d.	n.d.	‐	n.d.	n.d.	‐	‐	‐	‐
	24	n.d.	n.d.	‐	n.d.	n.d.	‐	n.d.	n.d.	n.d.	‐	n.d.	n.d.	‐	‐	‐	‐
Trimer AB	5	n.d.	n.d.	‐	n.d.	n.d.	‐	n.d.	n.d.	n.d.	‐	n.d.	n.d.	‐	‐	‐	‐
	24	n.d.	n.d.	‐	n.d.	n.d.	‐	n.d.	n.d.	n.d.	‐	n.d.	n.d.	‐	‐	‐	‐
Trimer BB	5	1.1 ± 0.2^a,b;**^	2.0 ± 0.6^c;**^	‐	2.5 ± 0.3^c;***^	n.d.	‐	1.9 ± 0.1^c;***^	n.d.	n.d.	‐	n.d.	4.2 ± 0.1^a^	‐	‐	‐	‐
	24	0.3 ± 0.2^c^	0.3 ± 0.1^c^	‐	0.1 ± 0.0^b^	2.1 ± 0.5^b^	‐	1.0 ± 0.2^b,c^	0.4 ± 0.1^c^	2.1 ± 0.3^c^	‐	0.6 ± 0.0^b^	68.6 ± 9.3^a;***^	‐	‐	‐	‐
Tetramers																	
Tetramer ABA	5	n.d.	n.d.	–	n.d.	n.d.	‐	n.d.	n.d.	n.d.	‐	n.d.	n.d.	‐	‐	‐	‐
	24	n.d.	n.d.	‐	n.d.	n.d.	‐	n.d.	n.d.	n.d.	‐	n.d.	n.d.	‐	‐	‐	‐
Tetramer BBB	5	0.2 ± 0.1^b^	0.2 ± 0.0^c^	‐	0.8 ± 0.1^c,d^	n.d.	‐	1.9 ± 0.4^c^	n.d.	n.d.	‐	n.d.	4.7 ± 0.6^a^	‐	‐	‐	‐
	24	1.5 ± 0.4^b;**^	5.7 ± 1.7^a;**^	‐	3.1 ± 1.4^a^	1.1 ± 0.6^b^	‐	3.6 ± 0.7^a,b;**^	n.d.	0.2 ± 0.1^c,d^	‐	0.2 ± 0.1^c^	19.6 ± 4.3^d;***^	‐	‐	‐	‐
Pentamer																	
Pentamer BBBB	5	0.1 ± 0.0^b^	0.1 ± 0.0^c^	‐	0.5 ± 0.0^d^	n.d.	‐	1.6 ± 0.1^c,d^	n.d.	n.d.	‐	n.d.	4.1 ± 0.8^a^	‐	‐	‐	‐
	24	2.0 ± 0.5^a; ***^	3.4 ± 0.3^b;**^	‐	3.6 ± 1.7^a;*^	0.9 ± 0.6^b^	‐	3.4 ± 0.2^a^	n.d.	0.3 ± 0.2^c,d^	‐	0.6 ± 0.3^b^	34.1 ± 1.6^c;***^	‐	‐	‐	‐
Galloyl derivatives																	
Monomer																	
EGCG	5	n.d.	n.d.	6.6 ± 0.1	n.d.	n.d.	1.4 ± 0.2	0.7 ± 0.2^c,d,e^	n.d.	n.d.	0.4 ± 0.0	n.d.	n.d.	3.9 ± 0.3	n.d.	20.2 ± 1.0^***^	‐
	24	n.d.	n.d.	n.d.	n.d.	n.d.	1.7 ± 0.2	n.d.	n.d.	n.d.	4.8 ± 1.4^**^	n.d.	n.d.	48.5 ± 7.5^***^	n.d.	12.2 ± 2.0^a^	‐
Dimer																	
T‐3′‐*O*‐G	5	‐	‐	‐	‐	‐	‐	‐	‐	‐	‐	‐	‐	‐	3.6 ± 0.7	n.d.	3.9 ± 0.8
	24	‐	‐	‐	‐	‐	‐	‐	‐	‐	‐	‐	‐	‐	17.5 ± 3.4^**^	5.8 ± 0.8^b^	8.1 ± 2.0^**^

Molar mass recoveries for catabolites were expressed as percentage with respect to the incubated concentration of parent compound (75 μmol L^1^ ^–^).

Data are expressed as mean ± SD (*n* = 3). n.d.: not detected. EGCG: (−)‐epigallocatechin‐3‐*O*‐gallate; T‐3′‐*O*‐G: theaflavin‐3′‐*O*‐gallate; OHPP‐2‐ol: 1‐(hydroxyphenyl)‐3‐(2″,4″,6″‐trihydroxyphenyl)‐propan‐2‐ol; 3′,5′‐DiOHPP‐2‐ol: 1‐(3′,5′‐dihydroxyphenyl)‐3‐(2″,4″,6″‐trihydroxyphenyl)‐propan‐2‐ol; 3′,4′‐DiOHPP‐2‐ol: 1‐(3′,4′‐dihydroxyphenyl)‐3‐(2″,4″,6″‐trihydroxyphenyl)‐propan‐2‐ol; 3′‐OH‐PVL: 5‐(3′‐hydroxyphenyl)‐γ‐valerolactone; 3′,5′‐DiOH‐PVL: 5‐(3′,5′‐dihydroxyphenyl)‐γ‐valerolactone; 3′,4′‐DiOH‐PVL: 5‐(3′,4′‐dihydroxyphenyl)‐γ‐valerolactone; 3′‐OH‐PVA: 5‐(3′‐hydroxyphenyl)valeric acid; 4‐OH‐(OH‐PVA): 4‐hydroxy‐5‐(hydroxyphenyl)valeric acid; 3′,5′‐DiOH‐PVA: 5‐(3′,5′‐dihydroxyphenyl)valeric acid; 3′,4′‐DiOH‐PVA: 5‐(3′‐4′‐dihydroxyphenyl)valeric acid; 3′‐OHPPA: 3‐(3′‐hydroxyphenyl)propanoic acid; 3′,5′‐DiOHPPA: 3‐(3′,5′‐dihydroxyphenyl)propanoic acid; 3,4,5‐TriOHBA: 3,4,5‐trihydroxybenzoic acid; TF: theaflavin. ‐ indicates catabolites which cannot be produced through the colonic degradation pathway of the incubated parent compound. Cell colors range from light to dark blue, indicating the range from low to high molar mass recovery for each catabolite over time: dark color is reported only when light color was indicated. Different lower case letters indicate significant differences among different fermented parent compounds considering the same incubation period (*p* < 0.05). *p < 0.05; **p < 0.01; ***p < 0.001, indicate significant differences comparing the same fermented substrate after different incubation period.

**Table 4:** Molar mass recoveries (%) for catabolites produced by interflavanic linkage fission and/or C‐ring opening of parent compounds having a DP >1 after 5 and 24 h faecal fermentation

Compounds	5 h	24 h
Dimers		
*Derived from catabolic pathway of dimer A2*		
1 fission dimer A2, form 1	27.1 ± 4.4	37.0 ± 1.1**
*Derived from catabolic pathway of dimer B2*		
1 fission dimer B2, form 1	7.3 ± 1.7	n.d.
2 fission dimer B2, form 1	n.d.	4.1 ± 0.7
Trimers		
*Derived from catabolic pathway of trimer AA*		
1 fission trimer AA, form 1	n.d.	1.8 ± 0.0
1 fission trimer AA, form 2	54.4 ± 8.9	112.8 ± 2.6***
*Derived from catabolic pathway of trimer AB*		
1 fission trimer AB, form 1	15.8 ± 2.4	25.6 ± 2.3***
1 fission trimer AB, form 2	23.4 ± 2.4	51.2 ± 3.6***
*Derived from catabolic pathway of trimer BB*		
1 fission trimer BB, form 1	11.7 ± 1.6	n.d.
2 fission trimer BB, form 1	2.7 ± 0.1	n.d.
Tetramers		
*Derived from catabolic pathway of tetramer BBB*		
1 fission tetramer BBB, form 1	3.3 ± 0.5***	1.1 ± 0.3
1 fission tetramer BBB, form 2	n.d.	2.0 ± 0.5
1 fission tetramer BBB, form 3	n.d.	1.2 ± 0.2
2 fission tetramer BBB, form 1	n.d.	14.9 ± 1.9
3 fission tetramer BBB, form 1	3.2 ± 0.5	n.d.
4 fission tetramer BBB, form 1	n.d.	3.1 ± 0.3

Molar mass recoveries for catabolites were expressed as percentage with respect to the incubated concentration of parent compound (75 μmol L^1^ ^–^). Data are expressed as mean ± SD (*n* = 3). n.d.: not detected; Cell colors range from light to dark blue, indicating the range from low to high molar mass recovery for each catabolite over time: dark color is reported only when light color was indicated. *p < 0.05; **p < 0.01; ***p < 0.001, indicate significant differences comparing the same fermented substrate after different incubation period.

#### 3.2.1 Diphenylpropan‐2‐ols and Fission Oligomers

Based on the incubated concentration of parent compound (75 µmol L^–1^), at 5 h, the molar mass recovery of 1‐(3′,4′‐dihydroxyphenyl)‐3‐(2″,4″,6″‐trihydroxyphenyl)‐propan‐2‐ol derived from (−)‐epicatechin incubation was significantly higher than after (+)‐catechin incubation (*p* < 0.001). A significant reduction of 1‐(3′,4′‐dihydroxyphenyl)‐3‐(2″,4″,6″‐trihydroxyphenyl)‐propan‐2‐ol molar mass recovery was observed in parallel with the increase of the DP of the precursors (dimer B2, trimer BB, tetramer BBB, pentamer) at 5 h incubation, whereas an opposite trend was observed at 24 h (Table 3), where the recovery of 1‐(3′,4′‐dihydroxyphenyl)‐3‐(2″,4″,6″‐trihydroxyphenyl)‐propan‐2‐ol increased with the DP of oligomers. After 5 h incubation, (+)‐catechin was more prone to produce 1‐(hydroxyphenyl)‐3‐(2″,4″,6″‐trihydroxyphenyl)‐propan‐2‐ol, a putative dehydroxylated derivative of 1‐(3′,4′‐dihydroxyphenyl)‐3‐(2″,4″,6″‐trihydroxyphenyl)‐propan‐2‐ol, than (−)‐epicatechin (*p* < 0.001). No significant differences (*p* > 0.05) emerged for the molar mass recoveries of 1‐(hydroxyphenyl)‐3‐(2″,4″,6″‐trihydroxyphenyl)‐propan‐2‐ol after B‐type oligomer incubation (dimer B2, trimer BB, tetramer BBB, pentamer) at 5 h, as well as between dimer B2 and trimer BB after 24 h incubation. Considering the different hydroxyl group pattern, a transient production of 1‐(3′,5′‐dihydroxyphenyl)‐3‐(2″,4″,6″‐trihydroxyphenyl)‐propan‐2‐ol was observed only after incubation of EGCG (Table 3).

Taking into account catabolites derived from fission of oligomeric parent compounds (Table 3), B‐type substrates (dimer B2, trimer BB, tetramer BBB) generally underwent various fission reactions with respect to their A and A‐B type counterparts (dimer A2, trimer AA, trimer AB), except for tetramer ABA where catabolism did not occur. Fission catabolites might be produced from interflavan cleavage and/or C‐ring opening (Figures S2 and S3, Supporting Information). The molar mass recovery for 1‐fission tetramer BBB (form 1) was significantly higher after 5 h compared to its concentration at 24 h (*p* < 0.001). Other cleavage catabolites such as 1‐fission dimer A2 (form 1), 1‐fission trimer AA (form 2), and 1‐fission trimer AB (forms 1 and 2) accumulated in significantly higher amounts after 24 h than after 5 h faecal incubation (Table 4).

#### 3.2.2 PVLs and PVAs

The molar mass recovery of 5‐(3′,4′‐dihydroxyphenyl)‐γ‐valerolactone produced from the faecal biotransformation of (−)‐epicatechin was 2.4‐ and 3.8‐fold higher than the quantity recovered after (+)‐catechin fermentation at 5 and 24 h incubation (*p* < 0.001 and *p* < 0.01), respectively (Table 3). Considering oligomers, the faecal incubation of dimer A2 led to the formation of 5‐(3′,4′‐dihydroxyphenyl)‐γ‐valerolactone, although in a significant lower amount than after dimer B2 incubation at 24 h (*p* < 0.001). 5‐(3′,4′‐Dihydroxyphenyl)‐γ‐valerolactone from dimer A2 was the sole 5C‐RFM identified after fermentation of A‐type oligomers. B‐type PACs (trimer BB, tetramer BBB, pentamer) were catabolized to 5‐(3′,4′‐dihydroxyphenyl)‐γ‐valerolactone (Table 3) regardless the DP and the incubation time. In contrast, 5‐(3′‐hydroxyphenyl)‐γ‐valerolactone, the 3′‐monohydroxylated product of 5‐(3′,4′‐dihydroxyphenyl)‐γ‐valerolactone, displayed no significant differences between the monomers (+)‐catechin and (−)‐epicatechin at 24 h, and it also accumulated after the 24 h incubation of all B‐type precursors (dimer B2, trimer BB, tetramer and BBB, pentamer).

Taking into account PVAs, although (−)‐epicatechin led to the highest molar mass recovery for 4‐hydroxy‐5‐(hydroxyphenyl)valeric acid and 5‐(3′,4′‐dihydroxyphenyl)valeric acid at 24 h, these catabolites were also produced after incubation of B‐type PCs, reaching similar recoveries after a 24 h incubation regardless the DP. 5‐(3′‐Hydroxyphenyl)valeric acid, a monohydroxylated catabolite derived putatively from both 4‐hydroxy‐5‐(hydroxyphenyl)valeric acid and 5‐(3′,4′‐dihydroxyphenyl)valeric acid, had the highest molar mass recovery when (−)‐epicatechin was fermented for 24 h, followed by (+)‐catechin, dimer B2 and trimer BB, for which a significant reduction was observed as the DP increased. Among galloyl derivatives, only EGCG was catabolized into two dihydroxylated PVLs, namely 5‐(3′,5′‐dihydroxyphenyl)‐γ‐valerolactone and 5‐(3′,4′‐dihydroxyphenyl)‐γ‐valerolactone, and one dihydroxylated PVA (5‐(3′,5′‐dihydroxyphenyl)valeric acid), the latter increasing 12‐fold at 24 h compared to the amount recovered after 5 h faecal incubation (*p* < 0.01) (Table 3). Stoichiometric balances for the production of 5C‐RFMs (PVLs and PVAs) calculated after the in vitro fermentation of each parent compound are reported in Table S3.

#### 3.2.3 Phenylpropanoic Acids

After the fermentation of 75 µmol L^–1^ of native compound, no significant differences were found in molar mass recovery for 3‐(3′‐hydroxyphenyl)propanoic acid derived from either (+)‐catechin or (−)‐epicatechin after a 5 h incubation or after 24 h when it was present in higher amounts (Table 3). 3‐(3′‐Hydroxyphenyl)propanoic acid was also one of the two low molecular weight catabolites recovered after dimer A2 faecal fermentation, together with 5‐(3′,4′‐dihydroxyphenyl)‐γ‐valerolactone, although its 24 h molar mass recovery was significantly lower compared to the amount recovered after dimer B2 catabolism (*p* < 0.001) (Table 3). Indeed, all B‐type substrates (dimer B2, trimer BB, tetramer BBB, pentamer) resulted in the production of 3‐(3′‐hydroxyphenyl)propanoic acid, after 5 h fermentation and accumulated in high amounts after 24 h. Trimer BB also had the highest molar mass recovery of 3‐(3′‐hydroxyphenyl)propanoic acid after 24 h. A second 3‐(hydroxyphenyl)propanoic acid, namely 3‐(3′,5′‐dihydroxyphenyl)propanoic acid, was produced exclusively by EGCG catabolism, and increased significantly over time with a 13.4‐fold increase at 24 h compared to 5 h (*p* < 0.001).

#### 3.2.4 Galloyl Derivatives of the Colonic Catabolism

The gallated substrates (EGCG and theaflavin‐3′‐*O*‐gallate) both yielded benzene‐1,2,3‐triol but only theaflavin‐3′‐*O*‐gallate yielded detectable 3,4,5‐trihydroxybenzoic acid, although it is presumed to be the precursor of the benzene‐1,2,3‐triol (Table 3). The significant (*p* < 0.001) greater yield of the triol from EGCG at 24 h is consistent. Neither was detected after incubation of the other substrates. Theaflavin was detected following degallation but (−)‐epigallocatechin was not.

### 3.3 Comprehensive Assessment of the Differences in the Catabolism of Flavan‐3‐ols Incubated

**Figure 2:**
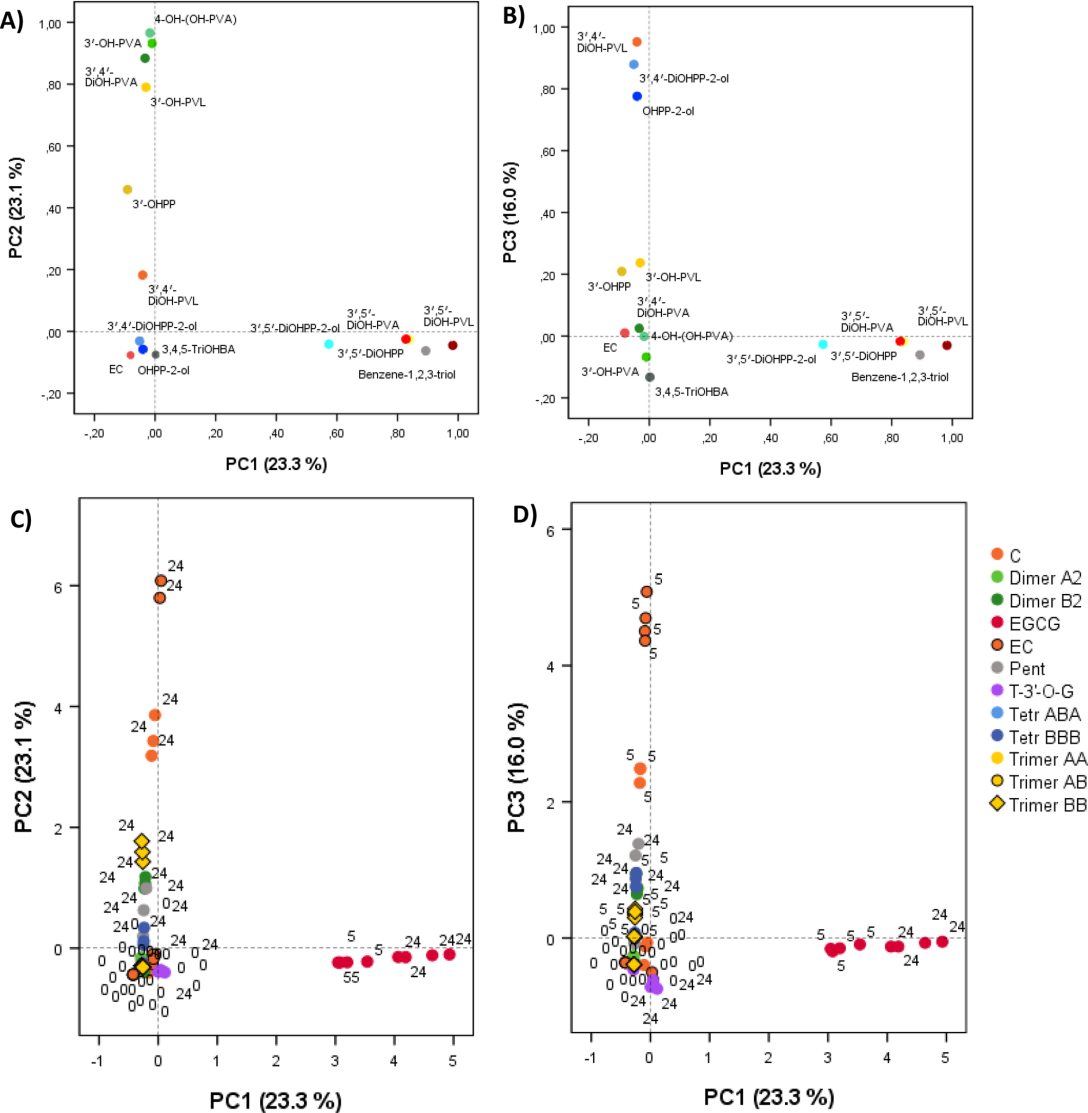
PCA to explore differences in the behavior of parent compounds in the in vitro colonic environment and in the appearance of catabolites over the faecal fermentation of monomeric and oligomeric flavan‐3‐ols. Loading plots of PC1 versus PC2 (A), PC1 versus PC3 (B). Score plots of the concentrations of identified catabolites obtained from PC1 and PC2 (C), PC1 and PC3 (D). The number accounts for the collection time (0, 5, and 24 h). C: (+)‐catechin; EC: (−)‐epicatechin; Dim: dimer; Trim: trimer; Tetr: tetramer; Pent: pentamer; EGCG: (−)‐epigallocatechin‐3‐*O*‐gallate; T‐3′‐*O*‐G: theaflavin‐3′‐*O*‐gallate. OHPP‐2‐ol: 1‐(hydroxyphenyl)‐3‐(2″,4″,6″‐trihydroxyphenyl)‐propan‐2‐ol; 3′,5′‐DiOHPP‐2‐ol: 1‐(3′,5′‐dihydroxyphenyl)‐3‐(2″,4″,6″‐trihydroxyphenyl)‐propan‐2‐ol; 3′,4′‐DiOHPP‐2‐ol: 1‐(3′,4′‐dihydroxyphenyl)‐3‐(2″,4″,6″‐trihydroxyphenyl)‐propan‐2‐ol; 3′‐OH‐PVL: 5‐(3′‐hydroxyphenyl)‐γ‐valerolactone; 3′,5′‐DiOH‐PVL: 5‐(3′,5′‐dihydroxyphenyl)‐γ‐valerolactone; 3′,4′‐DiOH‐PVL: 5‐(3′,4′‐dihydroxyphenyl)‐γ‐valerolactone; 3′‐OH‐PVA: 5‐(3′‐hydroxyphenyl)valeric acid; 4‐OH‐(OH‐PVA): 4‐hydroxy‐5‐(hydroxyphenyl)valeric acid; 3′,5′‐DiOH‐PVA: 5‐(3′,5′‐dihydroxyphenyl)valeric acid; 3′,4′‐DiOH‐PVA: 5‐(3′,4′‐dihydroxyphenyl)valeric acid; 3′‐OHPPA: 3‐(3′‐hydroxyphenyl)propanoic acid; 3′,5′‐DiOHPPA: 3‐(3′,5′‐dihydroxyphenyl)propanoic acid; 3,4,5‐TriOHBA: 3,4,5‐trihydroxybenzoic acid. Too specific compounds as fission catabolites from oligomers were not included.

A principal component analysis (PCA) including the concentrations of catabolites accumulating with most of the fermented substrates, namely diphenylpropan‐2‐ols (1‐(hydroxyphenyl)‐3‐(2″,4″,6″‐trihydroxyphenyl)‐propan‐2‐ol, 1‐(3′,4′‐dihydroxyphenyl)‐3‐(2″,4″,6″‐trihydroxyphenyl)‐propan‐2‐ol, 1‐(3′,5′‐dihydroxyphenyl)‐3‐(2″,4″,6″‐trihydroxyphenyl)‐propan‐2‐ol), 5C‐RFMs (5‐(3′‐hydroxyphenyl)‐γ‐valerolactone, 5‐(3′,4′‐dihydroxyphenyl)‐γ‐valerolactone, 5‐(3′,5′‐dihydroxyphenyl)‐γ‐valerolactone and 5‐(3′‐hydroxyphenyl)valeric acid, 4‐hydroxy‐5‐(hydroxyphenyl)valeric acid, 5‐(3′,4′‐dihydroxyphenyl)valeric acid, 5‐(3′,5′‐dihydroxyphenyl)valeric acid), 3‐(phenyl)propanoic acids (3‐(3′‐hydroxyphenyl)propanoic acid and 3‐(3′,5′‐dihydroxyphenyl)propanoic acid), benzoic acid and benzene derivatives (3,4,5‐trihydroxybenzoic acid and benzene‐1,2,3‐triol) and (−)‐epicatechin, revealed the existence of some differences regarding both the amounts of catabolites and the time of their appearance, that depend on the substrate incubated (**Figures** **2** and S1, Supporting Information). Three principal components (PCs) explained up to 62.6% of the total variance. The first PC (PC1) accounted for the 23.3% of the total variability (Figure 2A and B) and it was positively loaded mainly by 3′,5′‐dihydroxyphenyl catabolites (1‐(3′,5′‐dihydroxyphenyl)‐3‐(2″,4″,6″‐trihydroxyphenyl)‐propan‐2‐ol, 5‐(3′,5′‐dihydroxyphenyl)‐γ‐valerolactone, 5‐(3′,5′‐dihydroxyphenyl)valeric acid, and 3‐(3′,5′‐dihydroxyphenyl)propanoic acid) and a benzene derivative (benzene‐1,2,3‐triol). PC2, representing the 23.1% of the variance (Figure 2A and Figure S1, Supporting Information), was positively linked to 3′,4′‐dihydroxy and 3ʹ‐hydroxy “late‐products” of the colonic catabolism, such as some PVAs (5‐(3′‐hydroxyphenyl)valeric acid, 4‐hydroxy‐5‐(hydroxyphenyl)valeric acid, and 5‐(3′,4′‐dihydroxyphenyl)valeric acid), a 3‐(3′‐hydroxyphenyl)propanoic acid and a monohydroxylated PVL (5‐(3′‐hydroxyphenyl)‐γ‐valerolactone). In contrast, the third PC (PC3) accounted for the 16.0% of the total variability (Figure 2B and Supporting information Figure S1) and it was positively loaded mainly by “early products” of the catabolic pathway, such as diphenylpropan‐2‐ols (1‐(hydroxyphenyl)‐3‐(2″,4″,6″‐trihydroxyphenyl)‐propan‐2‐ol and 1‐(3′,4′‐dihydroxyphenyl)‐3‐(2″,4″,6″‐trihydroxyphenyl)‐propan‐2‐ol) and 5‐(3′,4′‐dihydroxyphenyl)‐γ‐valerolactone.

Score plots accounting for the catabolic pathway of each precursor are presented in Figure 2C and D. As expected, EGCG was characterized by the production of 3′,5′‐dihydroxyphenyl catabolites, as showed by PC1 scores, being distinct from the rest of the parent compounds (Figure 2C). Monomers elicited a well defined and different behavior in the production of phenolic catabolites in comparison to oligomers. Monomers were clearly distinguished considering the early and late products of their catabolic pathways: at 5 h, (−)‐epicatechin had the highest positive values for PC3 (5‐(3′,4′‐dihydroxyphenyl)‐γ‐valerolactone and diphenylpropan‐2‐ols), followed by (+)‐catechin (Figure 2D); at 24 h, (−)‐epicatechin was more prone to the production of late catabolites (PVAs, 3‐(3′‐hydroxyphenyl)propanoic acid, and 5‐(3′‐hydroxyphenyl)‐γ‐valerolactone) than (+)‐catechin (Figure 2C). This indicated that the catabolism of (−)‐epicatechin occurred at a faster rate than (+)‐catechin. On the other hand, if early products of the catabolic pathway of (−)‐epicatechin and (+)‐catechin (diphenylpropan‐2‐ols and 5‐(3′,4′‐dihydroxyphenyl)‐γ‐valerolactone) appeared at 5 h, these catabolites appeared in lower amounts, and later after B‐type oligomer incubation, as shown by PC2 and PC3 scores (Supporting Information Figure S1). Finally, the catabolism of PACs containing A‐type linkages was minimal as they showed values close to zero for all the PCs (Figure 2C and D).

### 3.4 Quantitative Profiles in the Production of Total 5‐Carbon Side Chain Ring Fission Catabolites and Total Microbial Catabolites

**Figure 3:**
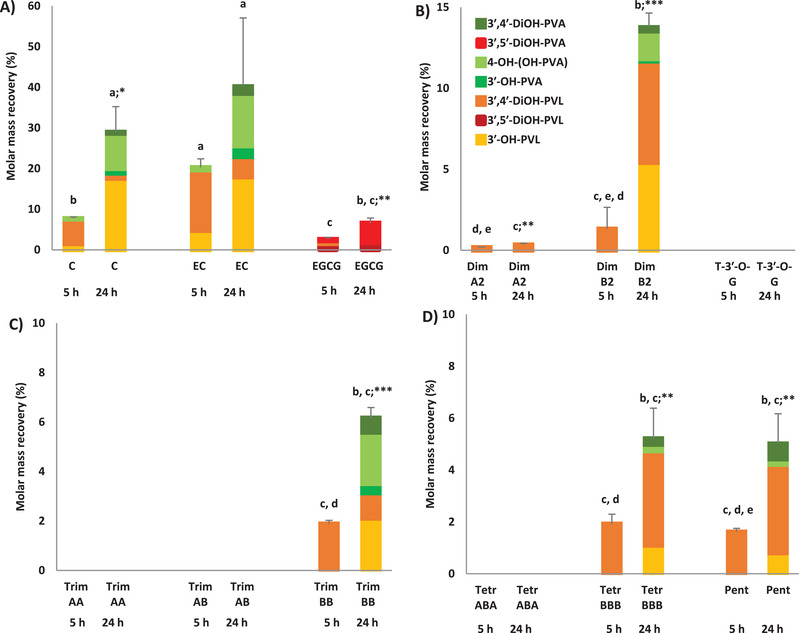
Molar mass recovery (%), reported as mean ± SD (*n* = 3), for the production of 5C‐RFCs (PVLs and PVAs) produced after 5 and 24 h faecal fermentation of C, EC and EGCG (A), dimers and T‐3′‐*O*‐G (B), trimers (C), tetramers and pentamer (D). Different lower case letters indicate significant differences among different fermented parent compounds considering the same incubation period (*p* < 0.05). *p < 0.05; **p < 0.01; ***p < 0.001 indicate significant differences comparing the same fermented substrate after different incubation period. Molar mass recoveries for catabolites were expressed as percentage with respect to the incubated concentration of parent compound (75 µmol L^–1^). C: (+)‐catechin; EC: (−)‐epicatechin; EGCG: (−)‐epigallocatechin‐3‐*O*‐gallate; Dim: dimer; T‐3′‐*O*‐G: theaflavin‐3′‐*O*‐gallate; Trim: trimer; Tetr: tetramer; Pent: pentamer; 3′,4′‐DiOH‐PVA: 5‐(3′,4′‐dihydroxyphenyl)valeric acid; 3′,5′‐DiOH‐PVA: 5‐(3′,5′‐dihydroxyphenyl)valeric acid; 4‐OH‐(OH‐PVA): 4‐hydroxy‐5‐(hydroxyphenyl)valeric acid; 3′‐OH‐PVA: 5‐(3′‐hydroxyphenyl)valeric acid; 3′,4′‐DiOH‐PVL: 5‐(3′,4′‐dihydroxyphenyl)‐γ‐valerolactone; 3′,5′‐DiOH‐PVL: 5‐(3′,5′‐dihydroxyphenyl)‐γ‐valerolactone; 3′‐OH‐PVL: 5‐(3′‐hydroxyphenyl)‐γ‐valerolactone

**Figure 4:**
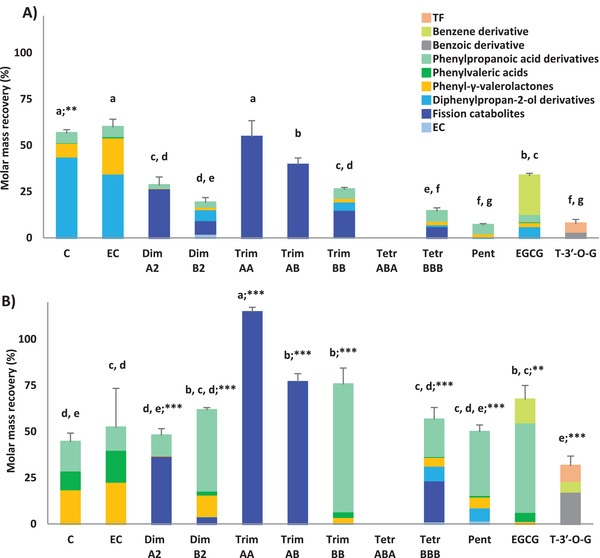
Molar mass recovery (%), reported as mean ± SD (*n* = 3), for the production of total catabolites produced after 5 (A) and 24 h (B) of faecal fermentation of monomeric and oligomeric flavan‐3‐ols. Different lower case letters indicate significant differences among different fermented parent compounds considering the same incubation period (*p* < 0.05). *p < 0.05; **p < 0.01; ***p < 0.001 indicate significant differences comparing the same fermented substrate after different incubation period. Molar mass recoveries for catabolites were expressed as percentage with respect to the incubated concentration of parent compound (75 µmol L^–1^). C: (+)‐catechin; EC: (−)‐epicatechin; Dim: dimer; Trim: trimer; Tetr: tetramer; Pent: pentamer; EGCG: (−)‐epigallocatechin‐3‐*O*‐gallate; T‐3′‐*O*‐G: theaflavin‐3′‐*O*‐gallate; TF: theaflavin. Diphenylpropan‐2‐ol derivatives include OHPP‐2‐ol, 3′,5′‐diOHPP‐2‐ol and 3′,4′‐diOHPP‐2‐ol. Fission catabolites include compounds produced by fission reaction of parent compounds having a DP above 1 (it might be either interflavan cleavage or C‐ring opening).

Significant main effects of treatment, time and treatment × time (*p* < 0.001) were found for the molar mass recovery calculated for total 5C‐RFCs, including PVLs and PVAs (**Figure** **3**) and for the sum of all quantified catabolites (**Figure** **4**), produced after 5 and 24 h faecal fermentation of parent compounds. Considering the incubated concentration, (+)‐catechin and (−)‐epicatechin led to the highest molar mass recoveries for total 5C‐RFCs at 5 h (7% and 20% for (+)‐catechin and (−)‐epicatechin, respectively) and 24 h (29% and 40% for (+)‐catechin and (−)‐epicatechin, respectively) of faecal incubation. Total PVLs and PVAs produced from EGCG incubation, beside the significant increase over the incubation process (*p* < 0.01), was in line with 5C‐RFCs produced after oligomer incubation (Figure 3A). Despite the absence of a significant difference between dimer A2 and dimer B2 at 5 h, the molar mass recovery for total 5C‐RFCs after dimer B2 fermentation was significantly higher than that derived from dimer A2 at the end of the incubation period (24 h, *p* < 0.01) (Figure 3B). The incubation of 75 µmol L^–1^ of B‐type oligomers did not lead to significant differences in total 5C‐RFC recovery after 5 and 24 h, which resulted ≃1.5% and ≃5.4%, respectively, for trimer BB, tetramer BBB, and pentamer BBBB (Figure 3C and D).

Considering the whole set of catabolites, most of the precursors led to a different set of catabolites, with some statistically significant differences in their overall recovery (Figure 4). Of note, trimer AA and trimer AB underwent fission without producing any low molecular weight phenolic catabolites (Figure 4). The molar mass recovery of total catabolites significantly increased over time for almost all the incubated parent flavan‐3‐ols, ranging from 6% to 59%, for pentamer and (+)‐catechin, respectively, at 5 h (Figure 4A), and from 31% to 114%, for theaflavin‐3′‐*O*‐gallate and trimer AA, respectively, at 24 h (Figure 4B).

**Figure 5:**
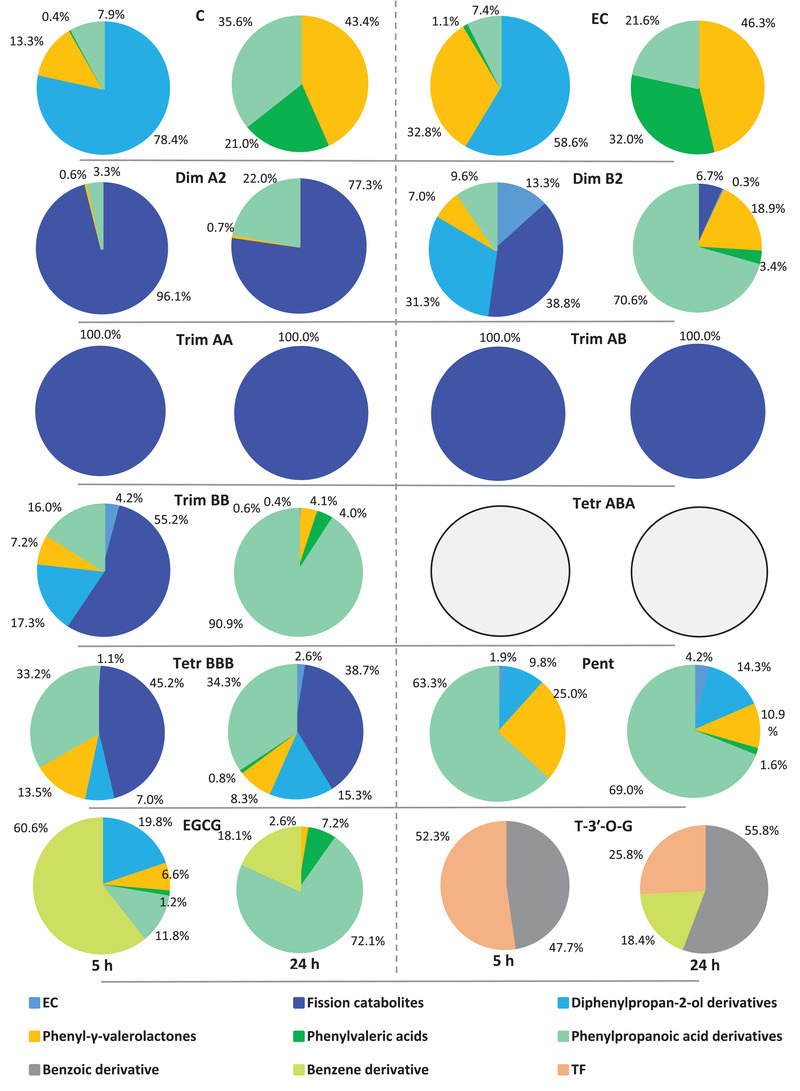
Relative contribution of catabolite classes after 5 and 24 h faecal fermentation of monomeric and oligomeric flavan‐3‐ols. C: (+)‐catechin; EC: (−)‐epicatechin; Dim: dimer; Trim: trimer; Tetr: tetramer; Pent: pentamer; EGCG: (−)‐epigallocatechin‐3‐*O*‐gallate; T‐3′‐*O*‐G: theaflavin‐3′‐*O*‐gallate; TF: theaflavin. Diphenylpropan‐2‐ol derivatives include OHPP‐2‐ol, 3′,5′‐diOHPP‐2‐ol and 3′,4′‐diOHPP‐2‐ol. Fission catabolites include compounds produced by fission reaction of parent compounds having a DP above 1 (it might be either interflavan cleavage or C‐ring opening).

The relative contribution of all the catabolite classes quantified after 5 and 24 h of faecal fermentation of different flavan‐3‐ols is showed in **Figure** **5**. Fission and diphenylpropan‐2‐ol derivatives were generally the most abundant catabolites produced at 5 h, ranging from 38% to 100%, and from 7% to 78%, respectively. With the exception of A‐type trimers, 3‐(3′‐hydroxyphenyl)propanoic acid was the most representative catabolite after a 24 h incubation, followed by 5C‐RFCs (in particular PVLs) (Figure 5).

## 4 Discussion

In the present study, a heterogeneous set of flavan‐3‐ol monomers, dimers and oligomers were fermented in vitro to elucidate the impact of the DP and nature of the inter‐flavan linkage on the production of colonic catabolites. Up to 32 flavan‐3‐ol catabolites were identified (Table 2), confirming that, except for tetramer ABA, all the incubated substrates underwent extensive catabolism. Limited fission of interflavan bonds was observed for substrates with only B‐type linkages consistent with previous reports.^[^ ^30^, ^34^, ^51^ ^]^

The major catabolic pathway is via reductive C‐ring cleavage, yielding diphenylpropan‐2‐ol derivatives.^[^ ^5^, ^52^ ^]^ The subsequent A‐ring cleavage led to PVLs and further PVAs by γ‐valerolactone ring opening,^[^ ^5^ ^]^ prior to dehydroxylation, primarily at the 4ʹ‐position of the B‐ring (Figure 1), followed by further oxidation steps. Diphenylpropan‐2‐ol derivatives were quantified both after monomer ((+)‐catechin, (−)‐epicatechin, and EGCG) and B‐type oligomer catabolism. The formation from tetramer BBB of three catabolites with masses 2 amu higher than the substrate suggests that at least three of the monomer units are susceptible to reductive C‐ring scission. In contrast, dimer B2 and trimer BB yielded only one such catabolite, but it is possible that some isomers coeluted. The formation from dimer B2 and trimer BB of catabolites having masses 4 amu higher than the substrate indicate that reductive C‐ring opening can occur twice in the same substrate. The catabolite 6 amu larger than trimer BB was sought but not found. The detection of catabolites with masses 4, 6, and 8 amu higher than the substrate in the tetramer BBB incubation demonstrates that for this substrate all four monomer units are susceptible, but, surprisingly, the pentamer did not yield any catabolites. In contrast for substrates with A‐type linkages (dimer A2, trimer AA, trimer AB) the equivalent catabolites had only increased by 2 amu indicating that only one unit had been reduced in each substrate, although the detection of two trimeric isomers indicates that at least two monomer units are accessible. Accordingly, catabolites 4 amu, and perhaps 6 amu higher, might have been detected if the incubations had continued beyond 24 h. The C‐ring opened catabolite of an (–)‐epicatechin‐A‐type dimer has been reported previously,^[^ ^27^, ^53^ ^]^ but so far as we are aware, this is the first report of such catabolites from an A‐type trimer and of catabolites produced from oligomeric PACs by reduction of more than two C‐rings (Table 4).

If monomers and end‐units of B‐type PCs mainly undergo the C‐ring fission through the activity of different colonic microbial strains,^[^ ^30^, ^50^, ^54^, ^55^ ^]^ A‐type PCs could potentially be subjected to C‐ring opening steps, as well as fission of interflavan bonds.^[^ ^27^, ^28^, ^53^ ^]^ However, it still remains unclear whether, prior to further C‐ring catabolism, the catabolic route of A‐type PCs involves only the interflavan bond fission or is also responsible for the possible formation a quinone methide derivatives through a rearrangement of the B‐ring after interflavan bond fission.^[^ ^28^, ^53^ ^]^ In contrast, no fission reactions beyond the loss of the galloyl moiety occurred in theaflavin‐3′‐*O*‐gallate faecal incubates.

Focusing on PVL and PVA production, although several studies reported the formation of these catabolites after monomer and B‐type dimer in vitro fermentation,^[^ ^5^ ^]^ it is evident that the structural configuration of the monomers (Figure 2), as well as the PC structure and the DP, affected the quantitative PVL and PVA profile. The highest molar mass recoveries for total 5C‐RFCs found with (+)‐catechin and (−)‐epicatechin (Figure 3) indicate that they were more efficiently converted into PVLs and PVAs than oligomers, in accordance with previous in vitro studies.^[^ ^31^, ^34^, ^50^ ^]^

The current study demonstrated that B‐type oligomers with high DP produce 5C‐RFCs. Stoupi and colleagues^[^ ^34^ ^]^ showed that the cleavage of the interflavan bond represents a minor pathway of the colonic catabolism of dimer B2, as no more than 10% was converted into (−)‐epicatechin in an in vitro faecal model. Since no significant differences were found in total PVL and PVA molar mass recovery in parallel with the increase of the DP of B‐type oligomers (less than 10%) (Figure 3), it seems likely that only a small fraction of monomeric units are released from the native structure, and become available for the C‐ring opening needed for the production of 5C‐RFMs. Previously, Stoupi and colleagues^[^ ^34^ ^]^ demonstrated that the lower unit of dimer B2 might be more easily accessible to colonic microbiota activity. Consequently, the main catabolic route of B‐type oligomers may involve direct ring fission of the terminal unit of oligomers, without any interflavan bond cleavage and monomer unit release. The low yield of C_6_‐C_5_ catabolites from oligomers compared with monomers and dimers could be due to the steric hindrance limiting access to the C‐ring. In keeping with this possibility it has been reported that no microbial catabolites are produced from 3‐methoxy‐(+)‐catechin intake.^[^ ^56^ ^]^

Regarding 5C‐RFM and A‐type PACs, this is the first study, to the best of our knowledge, to have demonstrated the production of 5‐(3′,4′‐dihydroxyphenyl)‐γ‐valerolactone following human faecal incubation of dimer A2. Recently, Chen et al.^[^ ^28^ ^]^ identified 5‐(3′,4′‐dihydroxyphenyl)‐γ‐valerolactone after in vitro fermentation of dimer A2 using rat faecal microbiota. However, there is an absence of information with human microbiota. The low molar mass recovery of total PVLs and PVAs calculated for dimer A2 compared to the dimer B2 (Figure 3) highlights that the additional ether C_2_‐C_7_ linkage strongly affects the C‐ring cleavage step in vitro. The DP also negatively influenced the colonic biotransformation of A‐type PCs. While dimer A2 catabolism yielded a few low molecular weight microbial catabolites, trimer AA and trimer AB underwent only a single fission‐catabolic reaction, whereas tetramer ABA was not catabolized by gut microbiota over the 24 h incubation period. An absence ring fission was also observed with theaflavin‐3′‐*O*‐gallate, that did not lead to PVLs or PVAs in accordance with previous reports.^[^ ^57^, ^58^ ^]^

From a stoichiometric point of view, the results of the present investigation indicate that the ingestion of ≈3 µmol of (+)‐catechin and (−)‐epicatechin or *ca*. 15 µmol of EGCG would potentially produce 1 µmol of circulating 5C‐RFMs (Table S3, Supporting Information). These quantities are in line with the estimation obtained for B‐type substrates: ≈7 and 18 µmol of dimer B2 and of the remaining B‐type substrates (trimer BB, tetramer BBB, pentamer), respectively, would be needed to achieve 1 µmol of PVL and PVA catabolites. In contrast, based on the reduced ability of gut microbiota to catabolize A‐type PCs, more than 290 µmol of dimer A2 are needed to produce 1 µmol of 5C‐RFMs. However, all (epi)catechin subunits of PAC oligomers are potentially available for microbial catabolism after microbial depolymerization. Consequently, assuming the full release of each (epi)catechin unit, in accordance with the DP (2‐5) of the native structure, the production of 1 µmol of 5C‐RFMs after the complete breakdown of oligomers drastically decreased (Table S3, Supporting Information), suggesting that oligomers may be a poor source of these C_6_‐C_5_ catabolites (Table S3, Supporting Information). In a human feeding trial with apple extracts containing flavan‐3‐ol monomers and oligomers (DP 2–10), Hollands et al.^[^ ^36^ ^]^ found that the 22% of ingested monomers were excreted as PVLs, whereas with oligomers there appeared to be negligible ring fission in vivo. Similarly, 202 µmol (representing the 26% of 24 h urinary phenolic catabolites) of PVLs and PVAs were excreted in urine following ingestion of Elstar apples containing 775 µmol of monomers.^[^ ^59^ ^]^ Following intake of cocoa flavan‐3‐ols, Ottaviani and colleagues^[^ ^26^ ^]^ pointed out that monomers represent a more significant source of PVLs than PCs, while Wiese and colleagues^[^ ^25^ ^]^ highlighted that the 24 h urinary excretion of 5‐(3′,4′‐dihydroxyphenyl)‐γ‐valerolactone represented, respectively, 6%, 4% and 0.7% of ingested (−)‐epicatechin, dimer B1 and oligomers with a mean DP of 5.9.

Among 3‐(phenyl)propanoic acids, derived from β‐oxidation of the side chain of PVAs,^[^ ^5^ ^]^ only 3‐(3′‐hydroxyphenyl)propanoic acid was quantified after monomer and PC fermentation. Appeldorn and colleagues^[^ ^30^ ^]^ found high amount of 3‐(3′‐hydroxyphenyl)propanoic acid following in vitro faecal incubation of B‐type dimers, suggesting a rapid conversion of PVAs into the mono‐hydroxylated 3‐(phenyl)propanoic acids. The limited number of C_6_‐C_2_ C_6_‐C_1_ and C_6_ gut microbiota catabolites detected in the present work beyond C_6_‐C_3_ 3‐(phenyl)propanoic acids could be due to the incubation periods: the fermentation stopped at 24 h, whereas some low molecular weight catabolites usually increase in later faecal incubation phases.^[^ ^27^, ^34^, ^35^ ^]^ Secondly, some low molecular weight catabolites have relatively poor MS ionization, resulting in a higher limit of detection and quantification (see Table S1, Supporting Information). The lack of some lower molecular weight catabolites could have partially influenced also the stoichiometric balance, since the molar mass recoveries calculated for total gut microbiota catabolites rarely reached the 100% (Figure 4). In addition, the quantification of some catabolites should be considered as semi‐quantification due to the absence of reference standards, possibly resulting in a mis‐estimation of their levels. Finally, although all the expected catabolites related to the known catabolic pathways of flavan‐3‐ols^[^ ^5^ ^]^ were monitored, some intermediates and/or unknown re‐arranged catabolites whose production was previously hypothesized,^[^ ^27^, ^28^, ^51^, ^55^ ^]^ might have increased the molar mass recovery for some compounds. Nevertheless, the present study opens new perspectives for an untargeted catabolomic approach addressing a complete, data‐driven investigation of the catabolites produced from PACs in the human colonic environment, as well as for NMR investigations to fully understand the structures of possible newly formed catabolites.

In conclusion, this study investigated for the first time the behavior of a variety of individual flavan‐3‐ol substrates in an in vitro human colonic environment. New catabolic routes, resulting in PC fission catabolites, have been established. The study sheds light on how structure can affect the interaction between the native flavan‐3‐ols and colonic microbial catabolic activity. The structural heterogeneity of native substrates strongly affected the profile of PVLs and PVAs produced during the in vitro fermentations. The calculated stoichiometric balances in the production of 5C‐RFMs could potentially support the experimental design of in vivo and in vitro models aiming at evaluating the catabolism of flavan‐3‐ols in different experimental settings (i.e., bioavailability and dose‐response studies, cell models, etc.). If catabolites are plausible candidates responsible for the recognized biological activity generally attributed to their native compounds,^[^ ^9^, ^11^, ^13^ ^]^ further studies are needed to fully clarify the I) catabolic fate of heterogeneous PAC sources and their ability to produce key bioactive catabolites, as well as other possible catabolites not yet identified, and II) which microbial populations might be involved in the catabolic pathway of oligomer flavan‐3‐ols in the colonic environment and thus, adding evidence on the type and interaction modes of these phytocompounds with the human gut microbiota.

## Conflict of Interest

The authors declare no conflict of interest.

## Author Contributions

G.D.P., L.B., D.D.R., and P.M. designed the study. G.D.P. conducted the study and performed the analysis. G.D.P., L.B., and P.M. analyzed and interpreted data. G.D.P. drafted the manuscript. L.B., P.M. F.B., M.N.C., A.C., and D.D.R. edited the manuscript. All authors critically read and approved the final version of the manuscript.

## Supporting information

Supporting Information

## Data Availability

The data that support the findings of this study are available from the corresponding author upon reasonable request.
