# Supplementary material for: In Vitro Faecal Fermentation of Monomeric and Oligomeric Flavan‐3‐ols: Catabolic Pathways and Stoichiometry
Source: Mol Nutr Food Res. 2022 Feb 18;66(21):2101090. doi: 10.1002/mnfr.202101090 (PMC9786279; doi:10.1002/mnfr.202101090)
Supplement: Supplementary file 1 — Supporting Information [file MNFR-66-2101090-s001.pdf]

Supporting information

***In vitro* faecal fermentation of monomeric and oligomeric flavan-3-ols: Catabolic pathways and stoichiometry**

Giuseppe Di Pede[1], Letizia Bresciani[1,]*, Furio Brighenti[1], Michael N. Clifford[2,3], Alan Crozier[4,5], Daniele Del Rio[1,6], Pedro Mena[1,6].

- [1]Human Nutrition Unit, Department of Food and Drug, University of Parma, Parma, Italy
- [2] School of Bioscience and Medicine, University of Surrey, Guildford, United Kingdom
- [3] Department of Nutrition, Dietetics and Food, Monash University, Notting Hill, Victoria, Australia
- [4] Department of Chemistry, King Saud University, Riyadh, Saudi Arabia
- [5] School of Medicine, Dentistry and Nursing, University of Glasgow, United Kingdom
- [6] Microbiome Research Hub, University of Parma, Parma, Italy

*Correspondence: Letizia Bresciani, Department of Food and Drugs, University of Parma; Medical School, Building C, Via Volturno, 39, 43125 Parma, Italy. letizia.bresciani@unipr.it

Table S1. Limit of detection (LOD) and limit of quantification (LOQ) (µmol L^{-1}) for reference standards used for the identification and quantification of gut microbiota catabolites. The nomenclature of gut microbiota catabolites was standardized according to Kay et al.[1]

Compound	LOD (µmol L^{-1})	LOQ (µmol L^{-1})
(+)-Catechin	0.02	0.05
(−)-Epicatechin	0.02	0.05
Dimer A2	0.02	0.05
Dimer B2	0.02	0.05
Trimer AA	0.02	0.05
Trimer AB	0.05	0.25
Trimer BB	0.05	0.25
Tetramer ABA	0.02	0.05
Tetramer BBB	0.05	0.25
Pentamer BBBB	0.05	0.25
(−)-Epigallocatechin-3-*O*-gallate	0.05	0.25
Theaflavin-3′-*O*-gallate	0.25	1.00
5-(4′-Hydroxyphenyl)-γ-valerolactone	0.06	1.12
5-(3′-Hydroxyphenyl)-γ-valerolactone	0.01	0.03
5-(3′,5′-Dihydroxyphenyl)-γ-valerolactone	0.02	0.06
5-(3′,4′-Dihydroxyphenyl)-γ-valerolactone	0.02	0.06
5-(3′,4′,5′-Trihydroxyphenyl)-γ-valerolactone	0.30	5.00
5-(4′-Hydroxyphenyl)valeric acid	0.25	5.00
3-Phenylpropanoic acid	0.25	2.00
3-(4′-Hydroxyphenyl)propanoic acid	2.00	5.00
3-(3′-Hydroxyphenyl)propanoic acid	2.00	5.00
3-(3′,4′-Dihydroxyphenyl)propanoic acid	0.25	2.00
Phenylacetic acid	0.05	50.00
4′-Hydroxyphenylacetic acid	2.00	5.00
3′-Hydroxyphenylacetic acid	10.00	25.00
3′,4′-Dihydroxyphenylacetic acid	0.05	2.00
Benzoic acid	2.00	5.00
4-Hydroxybenzoic acid	0.25	2.00
3-Hydroxybenzoic acid	0.25	2.00
3,4-Dihydroxybenzoic acid	0.05	0.25
3,4,5-Trihydroxybenzoic acid	0.05	0.25
4-Hydroxybenzaldehyde	0.05	0.25
Benzene-1,3,5-triol	0.25	2.00
Benzene-1,2,3-triol	0.25	2.00
3,4-Dihydroxybenzaldehyde	0.05	0.25

Table S2. Retention times and selective reaction monitoring (SRM) conditions for identification and quantification of monomeric and oligomeric flavan-3-ols and their gut microbiota catabolites by UHPLC-ESI-MS/MS. The nomenclature of gut microbiota catabolites was standardized according to Kay et al. [1]

Compound (Abbreviation)	RT (min)	Parent ion $(M - H)^-$ (*m/z*)	S-lens	Quantifier		Qualifier		Standard used for quantification
				Product ion (*m/z*)	CE (V)	Product ion (*m/z*)	CE (V)	
**Parent compounds**								
**Monomeric flavan-3-ols**								
(+)-Catechin	3.65	289	112	245	18	203	23	(+)-Catechin
(−)-Epicatechin	4.32	289	112	245	18	203	23	(−)-Epicatechin
**Oligomeric flavan-3-ols**								
**Dimers**								
Dimer A2	4.91	575	131	289	25	449	25	Dimer A2
Dimer B2	4.22	577	131	289	27	407	25	Dimer B2
**Trimers**								
Trimer AA	4.95	861	140	861	25	-	-	Trimer AA
Trimer AB	4.82	863	140	863	25	289	25	Trimer AB
Trimer BB	4.50	865	140	865	25	289	25	Trimer BB
**Tetramers**								
Tetramer ABA	5.05	574*	145	289	25	574	25	Tetramer ABA
Tetramer BBB	4.59	576*	145	289	22	125	42	Tetramer BBB
**Pentamer**								
Pentamer BBBB	4.68	720.6*	204	289	34	125	45	Pentamer BBBB
**Galloyl derivatives**								
**Monomer**								
(−)-Epigallocatechin-3-*O*-gallate (EGCG)	4.42	457	86	169	21	125	44	EGCG
**Dimer**								
Theaflavin-3′-*O*-gallate	5.60	715	98	563	30	715	30	Theaflavin-3′-*O*-gallate
**Fission catabolites**								
**Dimers**								
Derived from catabolic pathway of dimer A2								
1 Fission Dimer A2, form 1	5.18	577	131	577	25	291	25	Dimer A2

2 Fission Dimer A2, form 1	-	579	-	-	-	-	-	n.d.
Derived from catabolic pathway of dimer B2								
1 Fission Dimer B2, form 1	4.89	579	131	291	25	289	25	Dimer B2
2 Fission Dimer B2, form 1	5.53	581	131	581	25	291	25	Dimer B2
**Trimers**								
Derived from catabolic pathway of trimer AA								
1 Fission Trimer AA, form 1	5.06	863	140	863	25	-	-	Trimer AA
1 Fission Trimer AA, form 2	5.32	863	140	863	25	-	-	Trimer AA
2 Fission Trimer AA, form 1	-	865	-	-	-	-	-	n.d.
3 Fission Trimer AA, form 1	-	867	-	-	-	-	-	n.d.
Derived from catabolic pathway of trimer AB								
1 Fission Trimer AB, form 1	5.07	865	140	865	25	291	25	Trimer AB
1 Fission Trimer AB, form 2	5.17	865	140	865	25	291	25	Trimer AB
2 Fission Trimer AB, form 1	-	867	-	-	-	-	-	n.d.
3 Fission Trimer AB, form 1	-	869	-	-	-	-	-	n.d.
Derived from catabolic pathway of trimer BB								
1 Fission Trimer BB, form 1	4.71	867	140	867	25	291	25	Trimer BB
2 Fission Trimer BB, form 1	4.86	869	140	869	25	-	-	Trimer BB
3 Fission Trimer BB, form 1	-	871	-	-	-	-	-	n.d.
**Tetramers**								
Derived from catabolic pathway of tetramer ABA								
1 Fission Tetramer ABA, form 1	-	576*	-	-	-	-	-	n.d.
2 Fission Tetramer ABA, form 1	-	578*	-	-	-	-	-	n.d.
3 Fission Tetramer ABA, form 1	-	580*	-	-	-	-	-	n.d.
4 Fission Tetramer ABA, form 1	-	582*	-	-	-	-	-	n.d.
Derived from catabolic pathway of tetramer BBB								
1 Fission Tetramer BBB, form 1	4.80	578*	145	291	25	578	25	Tetramer BBB
1 Fission Tetramer BBB, form 2	5.60	578*	145	578	25	289	25	Tetramer BBB
1 Fission Tetramer BBB, form 3	6.21	578*	145	578	25	-	-	Tetramer BBB
2 Fission Tetramer BBB, form 1	5.54	580*	145	580	25	291	25	Tetramer BBB
3 Fission Tetramer BBB, form 1	4.63	582*	145	291	25	289	25	Tetramer BBB
4 Fission Tetramer BBB, form 1	5.23	584*	145	584	25	-	-	Tetramer BBB
**Pentamers**								

Derived from catabolic pathway of pentamer BBBB								
1 Fission Pentamer, form 1	-	722.6*	-	-	-	-	-	n.d.
2 Fission Pentamer, form 1	-	724.6*	-	-	-	-	-	n.d.
3 Fission Pentamer, form 1	-	726.6*	-	-	-	-	-	n.d.
4 Fission Pentamer, form 1	-	728.8*	-	-	-	-	-	n.d.
5 Fission Pentamer, form 1	-	730.6*	-	-	-	-	-	n.d.
**Theaflavin-3′-*O*-gallate derivatives**								
Derived from catabolic pathway of theaflavin								
1 Fission Theaflavin, form 1	-	565	-	-	-	-	-	n.d.
2 Fission Theaflavin, form 1	-	567	-	-	-	-	-	n.d.
Derived from catabolic pathway of theaflavin-3′-O-gallate								
1 Fission Theaflavin-3′-*O*-gallate	-	717	-	-	-	-	-	n.d.
2 Fission Theaflavin-3′-*O*-gallate	-	719	-	-	-	-	-	n.d.
**Diphenylpropan-2-ol derivatives**								
1-(Hydroxyphenyl)-3-(2″,4″,6″-trihydroxyphenyl)-propan-2-ol	4.84	275	98	231	16	191	30	3′,4′-diOH-PVL
1-(3′,5′-Dihydroxyphenyl)-3-(2″,4″,6″-trihydroxyphenyl)-propan-2-ol	4.16	291	98	123	30	167	30	3′,5′-diOH-PVL
1-(3′,4′-Dihydroxyphenyl)-3-(2″,4″,6″-trihydroxyphenyl)-propan-2-ol	4.36	291	98	123	30	247	16	3′,4′-diOH-PVL
1-(3′,4′,5′-Triihydroxyphenyl)-3-(2″,4″,6″-trihydroxyphenyl)-propan-2-ol	-	307	-	-	-	-	-	n.d.
1-(3′,4′,5′-Trihydroxyphenyl)-3-(2″,4″,6″-trihydroxyphenyl) propan-2-yl gallate[§]	-	459	-	-	-	-	-	n.d.
**Phenyl-γ-valerolactones**								
5-Phenyl-γ-valerolactone	-	175	-	-	-	-	-	n.d.
5-(4′-Hydroxyphenyl)-γ-valerolactone	4.92	191	67	147	16	106	31	n.q.
5-(3′-Hydroxyphenyl)-γ-valerolactone (3′-OH-PVL)	5.07	191	67	147	16	106	31	3′-OH-PVL
5-(3′,5′-Dihydroxyphenyl)-γ-valerolactone (3′,5′-diOH-PVL)	3.98	207	81	163	18	123	20	3′,5′-diOH-PVL
5-(3′,4′-Dihydroxyphenyl)-γ-valerolactone (3′,4′-diOH-PVL)	4.33	207	81	163	18	122	21	3′,4′-diOH-PVL
5-(3′,4′,5′-Trihydroxyphenyl)-γ-valerolactone	2.55	223	75	179	21	138	26	n.d.
**Phenylvaleric acids**								
5-Phenylvaleric acid	-	177	-	-	-	-	-	n.d.
5-(4′-Hydroxyphenyl)valeric acid	5.46	193	71	147	30	175	30	n.d.
5-(3′-Hydroxyphenyl)valeric acid	5.56	193	71	147	30	175	30	3′-OH-PVL
4-Hydroxy-5-(phenyl)valeric acid	-	193	-	-	-	-	-	n.d.
4-Hydroxy-5-(hydroxyphenyl)valeric acid	4.38	209	63	147	20	101	20	3′,4′-diOH-PVL
5-(3′,5′-Dihydroxyphenyl)valeric acid	4.75	209	63	191	15	147	20	3′,5′-diOH-PVL
5-(3′,4′-Dihydroxyphenyl)valeric acid	5.02	209	63	191	15	165	12	3′,4′-diOH-PVL
4-Hydroxy-5-(3′,5′-Dihydroxyphenyl)valeric acid	-	225	-	-	-	-	-	n.d.

4-Hydroxy-5-(3′,4′-Dihydroxyphenyl)valeric	-	225	-	-	-	-	-	n.d.
5-(3′,4′,5′-Trihydroxyphenyl)valeric acid	-	225	-	-	-	-	-	n.d.
4-Hydroxy-5-(3′,4′,5′-Trihydroxyphenyl)valeric acid	-	241	-	-	-	-	-	n.d.
**Phenylbutyric acids**								
4-Phenylbutyric acid	-	163	-	-	-	-	-	n.d.
4-(4′-Hydroxyphenyl)butyric acid	-	179	-	-	-	-	-	n.d.
4-(3′-Hydroxyphenyl)butyric acid	-	179	-	-	-	-	-	n.d.
4-(3′,4′-Dihydroxyphenyl)butyric acid	-	195	-	-	-	-	-	n.d.
4-(3′,5′-Dihydroxyphenyl)butyric acid	-	195	-	-	-	-	-	n.d.
4-(3′,4′,5′-Trihydroxyphenyl)butyric acid	-	211	-	-	-	-	-	n.d.
**Phenylpropanoic acid derivatives**								
3-Phenylpropanoic acid	5.64	149	60	149	10	105	13	n.d.
3-(4′-Hydroxyphenyl)propanoic acid	4.37	165	64	121	13	93	14	n.d.
3-(3′-Hydroxyphenyl)propanoic acid (3′-OHPPA)	4.65	165	64	121	13	119	18	3′-OHPPA
2-Hydroxy-3-(phenyl)propanoic acid	-	165	-	-	-	-	-	n.d.
3-(3′,5′-Dihydroxyphenyl)propanoic acid	2.58	181	64	137	14	-	-	3′,4′-diOHPPA
3-(3′,4′-Dihydroxyphenyl)propanoic acid (3′,4′-diOHPPA)	3.27	181	64	137	14	109	18	n.d.
2-Hydroxy-3-(hydroxyphenyl)propanoic acid	-	181	-	-	-	-	-	n.d.
3-(4′-Hydroxy-3′-methoxyphenyl)propanoic acid	-	195	-	-	-	-	-	n.d.
3-(3′,4′,5′-Trihydroxyphenyl)propanoic acid	-	197	-	-	-	-	-	n.d.
**Phenylacetic acid derivatives**								
Phenylacetic acid	5.04	135	40	91	10	135	10	n.d.
4′-Hydroxyphenylacetic acid	3.14	151	75	107	13	-	-	n.d.
3′-Hydroxyphenylacetic acid	3.79	151	75	107	13	-	-	n.d.
3′,4′-Dihydroxyphenylacetic acid	1.85	167	35	123	13	122	28	n.d.
3′,4′,5′-Trihydroxyphenylacetic acid	-	183	-	-	-	-	-	n.d.
**Benzoic acid derivatives**								
Benzoic acid	4.98	121	68	77	13	121	10	n.d.
4-Hydroxybenzoic acid	2.73	137	72	93	16	-	-	n.d.
3-Hydroxybenzoic acid	3.62	137	72	93	16	-	-	n.d.
3,4-Dihydroxybenzoic acid	1.62	153	74	109	16	108	26	n.d.
3,5-Dihydroxybenzoic acid	-	153	-	-	-	-	-	n.d.
3,4,5-Trihydroxybenzoic acid (3,4,5-triOHBA)	0.96	169	78	125	17	79	25	3,4,5-triOHBA
**Benzene derivatives**								
Benzene-1,2-diol	-	109	-	-	-	-	-	n.d.

4-Hydroxybenzaldehyde	3.70	121	68	92	26	120	20	n.d.
Hydroxybenzyl alcohol	-	123	-	-	-	-	-	n.d.
Benzene-1,3,5-triol	0.88	125	62	125	10	57	17	n.d.
Benzene-1,2,3-triol	1.06	125	62	125	10	79	22	benzene-1,2,3-triol
3,4-Dihydroxybenzaldehyde	2.34	137	72	136	22	108	26	n.d.
2,4,6-Trihydroxybenzaldeyde	-	153	-	-	-	-	-	n.d.
**Monomer units**								
(−)-Epigallocatechin	-	305	-	-	-	-	-	n.d.
(−)-Epicatechin-3-*O*-gallate	-	441	-	-	-	-	-	n.d.
**Dimer units**								
Theanaphthoquinone[#]	-	533	-	-	-	-	-	n.d.
Theaflavin	5.45	563	98	241	30	269	30	Theaflavin-3′-*O*-gallate

RT = retention time; *m/z* = mass to charge ratio; CE = collision energy; n.d. = not detected; n.q. = not quantified because below the LOQ. [§]catabolite previously quantified after faecal fermentation of EGCG[2]; [~]catabolite previously quantified after faecal fermentation of EGCG [3]; [#]catabolite previously quantified after faecal fermentation of theaflavin-3,3′-digallate[2]. * Compounds were monitored as $[M-2H]^{2-}$.

Table S3. Stoichiometric balances in the production of total 5C-RFMs (PVLs and PVAs) from each parent compound fermented *in vitro*.

Compound	24 h MMR for total 5C-RFMs * (%)	Quantity of parent compound to achieve 1 µmol of total 5C-RFMs (µmol)	24 h MMR for total 5C-RFMs § (%)	Quantity of parent compound to achieve 1 µmol of total 5C-RFMs after complete breakdown of each monomeric subunit (µmol)
**Monomeric flavan-3-ols**				
(+)-Catechin	29.0	3.5	29.0	3.5
(−)-Epicatechin	40.2	2.5	40.2	2.5
**Oligomeric flavan-3-ols**				
**Dimers**				
Dimer A2	0.3	295.3	0.2	590.5
Dimer B2	13.7	7.3	6.9	14.5
**Trimers**				
Trimer AA	0.0	0.0	0.0	0.0
Trimer AB	0.0	0.0	0.0	0.0
Trimer BB	6.2	16.2	2.1	48.7
**Tetramers**				
Tetramer ABA	0.0	0.0	0.0	0.0
Tetramer BBB	5.2	19.2	1.3	76.8
**Pentamer**				
Pentamer BBBB	5.0	20.0	1.0	99.8
**Galloyl derivatives**				
**Monomer**				
(−)-Epigallocatechin-3-*O*-gallate	6.6	15.2	6.6	15.2
**Dimer**				
Theaflavin-3′-*O*-gallate	0.0	0.0	0.0	0.0

MMR: molar mass recovery;
*MMR calculated assuming the production of 5C-RFMs from 1 µmol of incubated parent compound;
§MMR calculated assuming the production of 5C-RFMs from 1 µmol of all the possible monomeric unit released from the oligomeric structure in accordance with the DP (2-5) of parent compound.

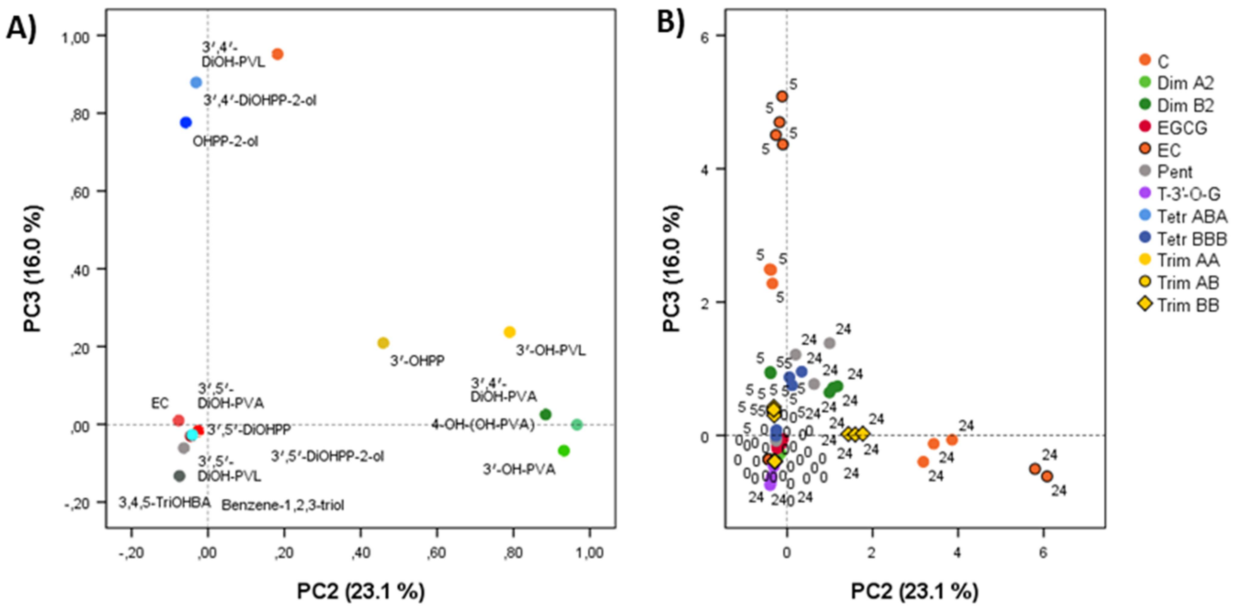

Figure S1. Principal component analysis (PCA) to explore differences in the behavior of parent compounds in the *in vitro* colonic environment and in the appearance of gut microbiota catabolites over the faecal fermentation of monomeric and oligomeric flavan-3-ols. Loading plots of PC2 versus PC3 (**A**). Score plots of the concentrations of identified gut microbiota catabolites obtained from PC2 and PC3 (**B**). The number accounts for the collection time (0 h, 5 h and 24 h). C: (+)-catechin; EC: (−)-epicatechin; Dim: dimer; Trim: trimer; Tetr: tetramer; Pent: pentamer; EGCG: (−)-epigallocatechin-3-*O*-gallate; T-3′-O-G: theaflavin-3′-*O*-gallate. OHPP-2-ol: 1-(hydroxyphenyl)-3-(2″,4″,6″-trihydroxyphenyl)-propan-2-ol; 3′,5′-DiOHPP-2-ol: 1-(3′,5′-dihydroxyphenyl)-3-(2″,4″,6″-trihydroxyphenyl)-propan-2-ol; 3′,4′-DiOHPP-2-ol: 1-(3′,4′-dihydroxyphenyl)-3-(2″,4″,6″-trihydroxyphenyl)-propan-2-ol; 3′-OH-PVL: 5-(3′-hydroxyphenyl)-γ-valerolactone; 3′,5′-DiOH-PVL: 5-(3′,5′-dihydroxyphenyl)-γ-valerolactone; 3′,4′-DiOH-PVL: 5-(3′,4′-dihydroxyphenyl)-γ-valerolactone; 3′-OH-PVA: 5-(3′-hydroxyphenyl)valeric acid; 4-OH-(OH-PVA): 4-hydroxy-5-(hydroxyphenyl)valeric acid; 3′,5′-DiOH-PVA: 5-(3′,5′-dihydroxyphenyl)valeric acid; 3′,4′-DiOH-PVA: 5-(3′,4′-dihydroxyphenyl)valeric acid; 3′-OHPP: 3-(3′-hydroxyphenyl)propanoic acid; 3′,5′-DiOHPP: 3-(3′,5′-dihydroxyphenyl)propanoic acid; 3,4,5-TriOHBA: 3,4,5-trihydroxybenzoic acid. Too specific compounds as fission catabolites from oligomers were not included.

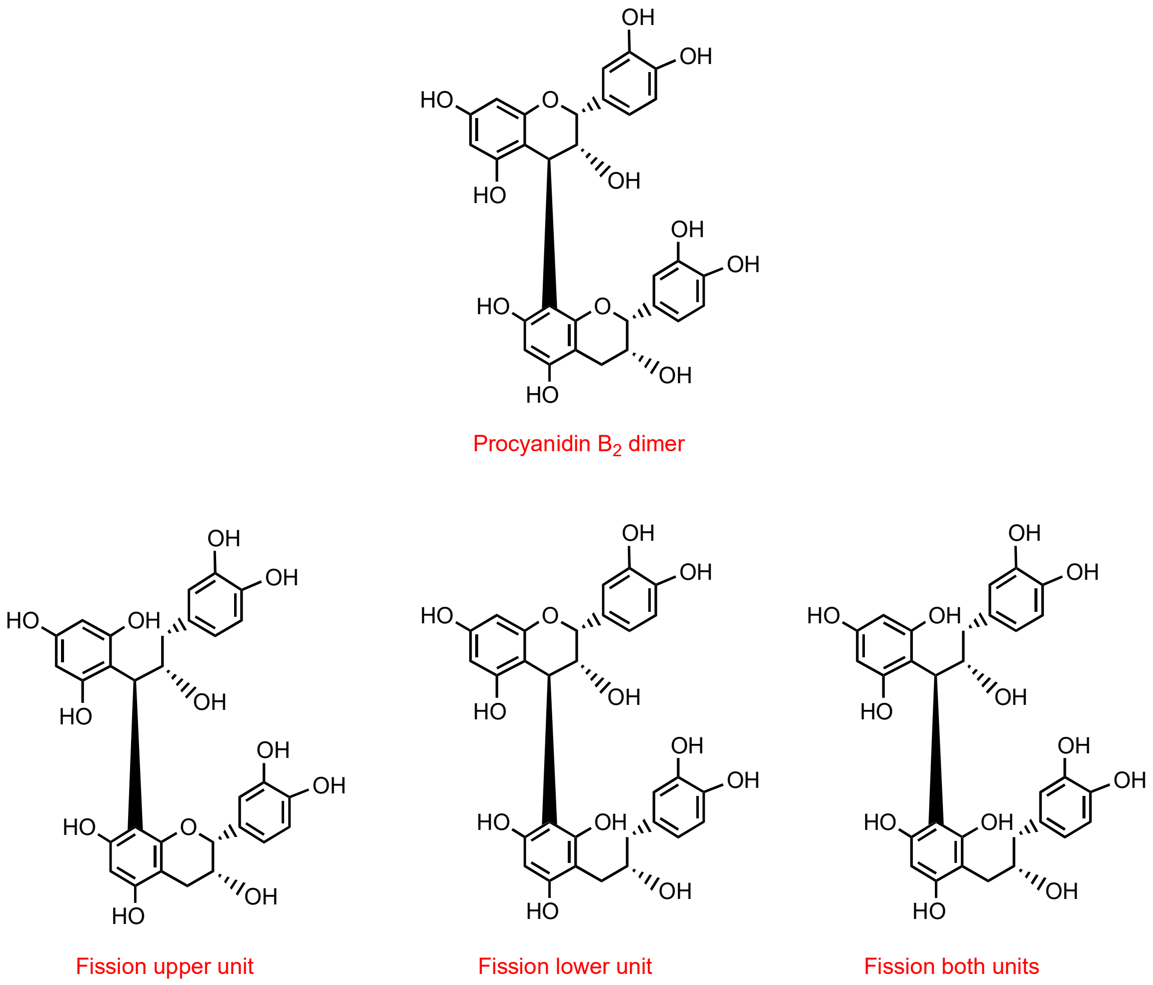

Figure S2. Fission derivatives of dimer B2

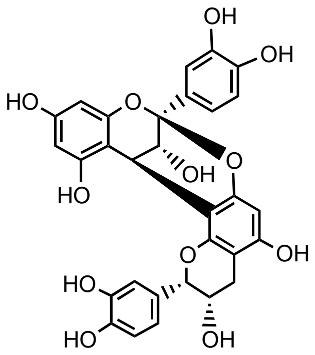

Procyanidin A_2 dimer

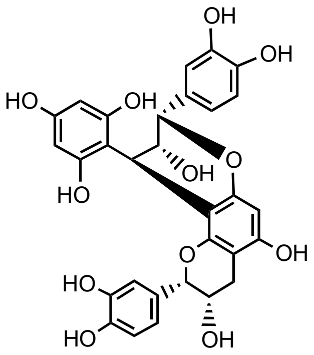

Fission upper unit

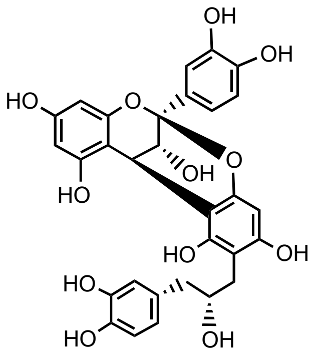

Fission lower unit

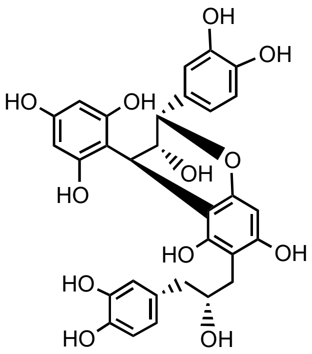

Fission both units

Figure S3. Fission derivatives of dimer A2

References

[1] C. D. Kay, M. N. Clifford, P. Mena, G. J. McDougall, C. Andres-Lacueva, A. Cassidy, D. Del Rio, N. Kuhnert, C. Manach, G. Pereira-Caro, A. Rodriguez-Mateos, A. Scalbert, F. Tomás-Barberán, G. Williamson, D. S. Wishart, A. Crozier, *Am. J. Clin. Nutr.* **2020**, *112*, 1051–1068.

[2] Z. Liu, W. J. C. de Bruijn, M. E. Bruins, J.-P. Vincken, *J. Agric. Food Chem.* **2020**, *69*, 232–245.

[3] Z. Liu, W. J. C. de Bruijn, M. E. Bruins, J.-P. Vincken, *J. Agric. Food Chem.* **2020**, *68*, 9804–9815.
